# Supramolecular Engineering of Vinylene‐Linked Covalent Organic Framework – Ruthenium Oxide Hybrids for Highly Active Proton Exchange Membrane Water Electrolysis

**DOI:** 10.1002/adma.202417374

**Published:** 2025-02-03

**Authors:** Kexin Wang, Shunqi Xu, Dashuai Wang, Zhenhui Kou, Yubin Fu, Michał Bielejewski, Verónica Montes‐García, Bin Han, Artur Ciesielski, Yang Hou, Paolo Samorì

**Affiliations:** ^1^ Key Laboratory of Biomass Chemical Engineering of Ministry of Education College of Chemical and Biological Engineering Zhejiang University Hangzhou 310 027 China; ^2^ University of Strasbourg CNRS ISIS UMR 7006, 8 Allée Gaspard Monge Strasbourg F‐67000 France; ^3^ School of Energy and Environment Southeast University Nanjing 211 189 China; ^4^ Institute of Zhejiang University‐Quzhou Quzhou 324 000 China; ^5^ Chair of Molecular Functional Materials Center for Advancing Electronics Dresden (cfaed) and Faculty of Chemistry and Food Chemistry Technische Universität Dresden Mommsenstrasse 4 0 1069 Dresden Germany; ^6^ Institute of Molecular Physics Polish Academy of Sciences M. Smoluchowskiego 17 Poznan 60–179 Poland; ^7^ Zhejiang University Hydrogen Energy Institute Hangzhou 310 027 China

**Keywords:** vinylene‐linked covalent organic frameworks, oriented hydrogen‐bonded network, proton exchange membrane water electrolysis, ruthenium dioxides, stabilized transition state of oxo‐intermediates

## Abstract

The controlled formation of a functional adlayer at the catalyst‐water interface is a highly challenging yet potentially powerful strategy to accelerate proton transfer and deprotonation for ultimately improving the performance of proton‐exchange membrane water electrolysis (PEMWE). In this study, the synthesis of robust vinylene‐linked covalent organic frameworks (COFs) possessing high proton conductivities is reported, which are subsequently hybridized with ruthenium dioxide yielding high‐performance anodic catalysts for the acidic oxygen evolution reaction (OER). In situ spectroscopic measurements corroborated by theoretical calculations reveal that the assembled hydrogen bonds formed between COFs and adsorbed oxo‐intermediates effectively orient interfacial water molecules, stabilizing the transition states for intermediate formation of OER. This determines a decrease in the energy barriers of proton transfer and deprotonation, resulting in exceptional acidic OER performance. When integrated into a PEMWE device, the system achieves a record current density of 1.0 A cm^−2^ at only 1.54 V cell voltage, with a long‐term stability exceeding 180 h at industrial‐level 200 mA cm^−2^. The approach relying on the self‐assembly of an oriented hydrogen‐bonded adlayer highlights the disruptive potential of COFs with customizable structures and multifunctional sites for advancing PEMWE technologies.

## Graphical abstract

A functionalized vinylene‐linked covalent organic framework adlayer is constructed to precisely tailor the orientation of interfacial water molecule to stabilize the transition state for intermediates adsorption/desorption, optimizing the proton exchange membrane water electrolysis performance.

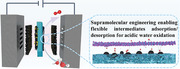

## 1 Introduction

**Figure 1:**
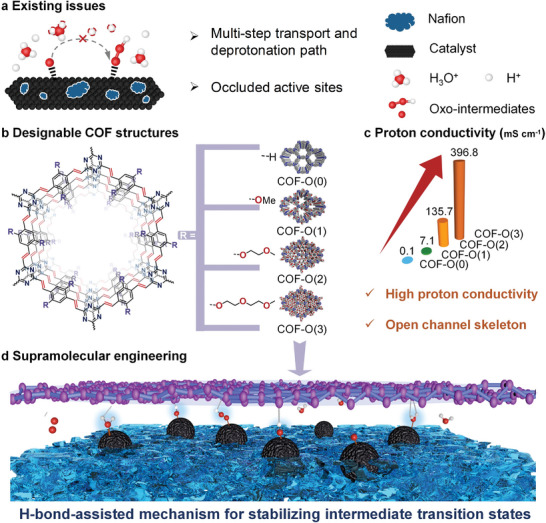
a) Challenges in the traditional catalytic system for acidic OER. b) The structural models of COF‐O(n), where n = 0, 1, 2, and 3 correspond to the ethoxy side chains of varying lengths. Red denotes oxygen (O); blue represents nitrogen (N); gray indicates carbon (C), and white shows hydrogen (H). c) Proton conductivity at 80 °C of COF‐O(n). d) Schematic illustration of a hydrogen‐bonded network at the catalyst‐water interface during the transformation of intermediates for high performance PEM device.

Proton‐exchange membrane water electrolysis (PEMWE) represents a promising technology for achieving rapid response and high‐energy efficiency in sustainable production of green hydrogen.^[^ ^1^ ^]^ However, the large‐scale implementation of PEMWE is primarily limited by the anodic oxygen evolution reaction (OER), which suffers from high catalytic overpotential during the four‐step proton‐coupled electron transfer deprotonation process.^[^ ^2^ ^]^ To date, significant efforts have been devoted to modulating electronic structures,^[^ ^3^ ^]^ controlling coordination environment,^[^ ^4^ ^]^ and regulating adsorption pathways^[^ ^5^ ^]^ to enhance OER performance. However, less attention has been paid to accelerating the deprotonation process of reaction intermediates and protecting active sites from being buried by linking polymers (e.g., Nafion), despite the critical importance of both factors (**Figure** **1**a).^[^ ^6^ ^]^ The dehydrogenation of adsorbed oxo‐intermediates is confined at the water‐hydronium interface due to the excessive concentration of protons in acidic electrolytes.^[^ ^7^ ^]^ Toward this end, the formation of a hydrogen bonded network at catalyst‐reactant interface is essential for providing optimal orientations to reactive hydrogen atoms,^[^ ^8^ ^]^ thereby improving the kinetics of hydrogen electrocatalysis.^[^ ^9^ ^]^ However, due to the non‐covalent nature of these interactions, achieving a precise control and stability over the hydrogen‐bond orientations at the water‐catalyst interface remains a significant challenge.^[^ ^10^ ^]^

Porous crystalline materials are known to offer various hydrogen‐bonding configurations owing to their chemical diversity and abundance of functional units.^[^ ^11^ ^]^ Among them, covalent organic frameworks (COFs) are an emerging class of crystalline porous polymers featuring tunable structures, large surface areas,^[^ ^12^ ^]^ and open pore channels.^[^ ^13^ ^]^ These characteristics make COFs an ideal platform for adjusting proton transfer mechanisms through the incorporation of hydrogen‐bonding sites.^[^ ^14^ ^]^ When combined with catalytically active materials, COFs could link ionomers and catalysts to simultaneously tailor the three‐phase interface and expose additional active sites, thus enhancing the performance of the electrochemical device.^[^ ^15^ ^]^ Nevertheless, the potential of COFs containing hybrids with an intermediate sandwich structure to create an oriented hydrogen‐bonded network for boosting OER electrocatalysis remains unexplored.

In this work, we meticulously constructed a proton‐transfer channel layer by using a series of vinylene‐linked COFs equipped with ethoxy side chains of varying lengths (referred to as COF‐O(n), n = 0, 1, 2, and 3) (Figure 1b). These COFs were then integrated with a standard OER catalyst (i.e., ruthenium dioxide, RuO_2_) to stabilize critical intermediate transition states through optimized hydrogen‐bonded orientations, yielding an exceptional OER performance. The periodically conjugated skeletons with robust vinylene linkages exhibited remarkable stability under strongly acidic conditions. The predesigned hydrophilic functional groups facilitated high proton conductivity, reaching up to 0.396 S cm^−1^, and enabled controllable proton transfer through the Grotthuss mechanism (Figure 1c). In situ spectroscopies combined with density functional theory (DFT) calculations revealed that the hydrogen‐bond interactions established by the engineered COF layers stabilized the transition states of oxo‐intermediates, thereby facilitating the kinetics of proton transfer and deprotonation (Figure 1d). Additionally, theoretical calculations provided unambiguous evidence that the hybridization of RuO_2_ with COFs is a viable route to lower the energy barrier for the rate‐determining step (*O + H_2_O → *OOH + H^+^ + e^−^). Significantly, our PEMWE setup demonstrated a low cell voltage of 1.54 V at current densities up to 1.0 A cm^−2^, surpassing the performance of commercial RuO_2_ catalyst.

## 2 Results and Discussion

### 2.1 Synthesis and Characterizations

**Figure 2:**
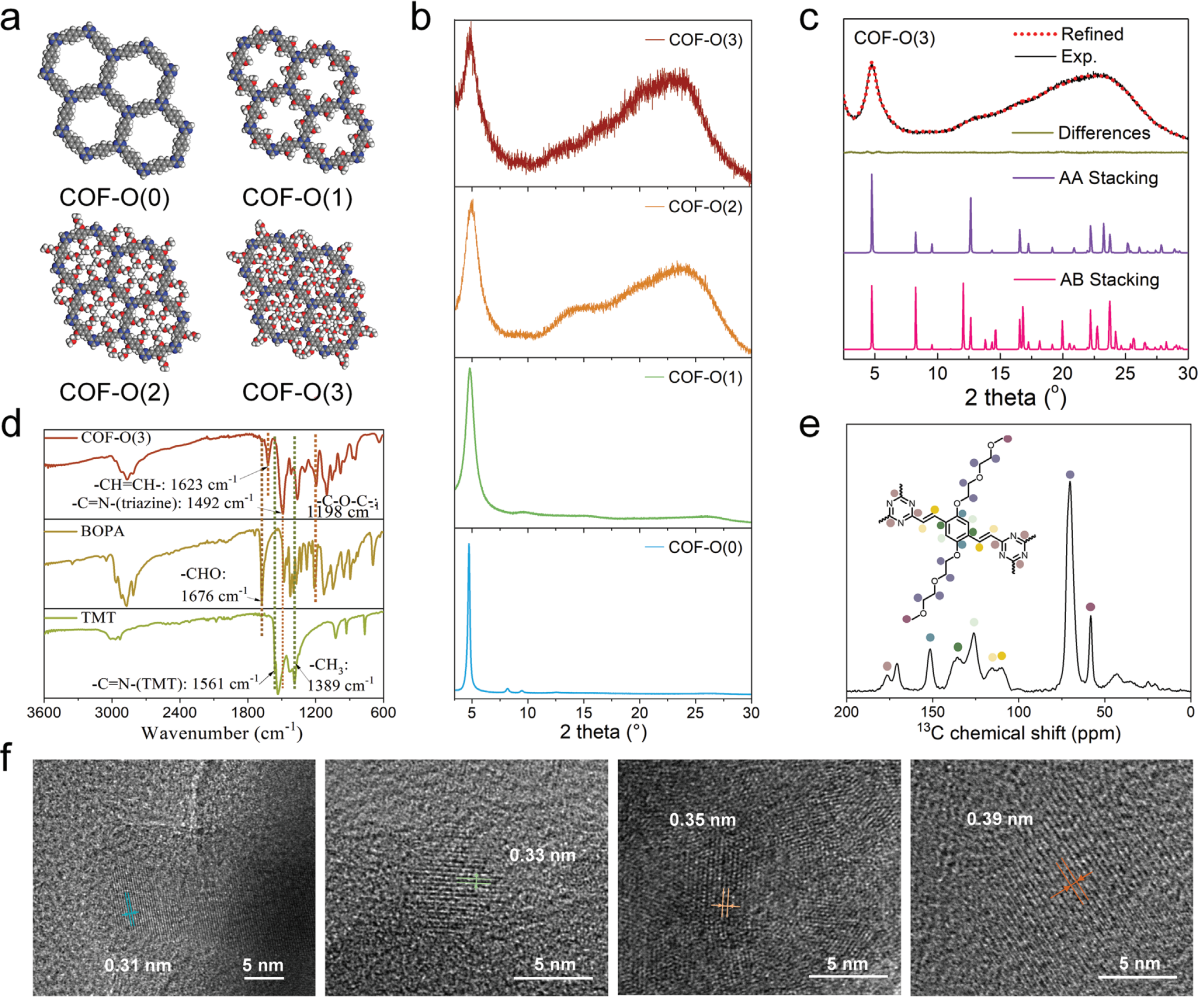
a) Structural models and b) XRD patterns for COF‐O(n), where n = 0, 1, 2, and 3. c) Pawley refined pattern (black), experimental pattern (red), differences (green), simulated eclipsed (AA) stacking pattern (purple), and simulated eclipsed (AB) stacking pattern (pink). d) FT‐IR spectra of COF‐O(3) and corresponding BOPA and TMT monomers. e) ^13^C CP/MAS NMR spectrum of COF‐O(3). f) HRTEM images of COF‐O(n) (where n = 0, 1, 2, and 3) are presented sequentially from left to right, with the corresponding layer‐to‐layer distances indicated in the images.

To leverage the robust properties of sp^2^ carbon conjugation,^[^ ^16^ ^]^ we functionalized the vinylene‐linked COF scaffold with diverse ethoxy side chains differing in their length, thus content of oxygen atoms (**Figure** **2**a). Such side chains have been designed to act as hydrogen‐bond acceptors. Experimental and simulated powder X‐ray diffraction (PXRD) studies were conducted to confirm the formation of the desired COFs. The PXRD patterns of COF‐O(n) (n = 0, 1, 2, and 3) displayed characteristic peaks at 4.7‐4.8°, corresponding to the (100) plane (Figure 2b). These (100) diffraction peaks, along with secondary reflections, confirmed the successful synthesis of highly crystalline COFs. PXRD pattern simulations using Materials Studio indicated that these COFs adopt a preferred eclipsed stacking arrangement characterized by the lowest total energies (Figure 2c; Figure S1 and Tables S1‐S4, Supporting Information).

The structures of synthesized COFs were further confirmed by Fourier transform infrared (FT‐IR) and ^13^C cross‐polarization/magic angle spinning solid‐state nuclear magnetic resonance (^13^C CP/MAS ssNMR) spectroscopies. The FT‐IR spectra displayed characteristic bands for C = C and C = N (in triazine) stretching modes at ca. 1623 and 1492 cm^−1^, respectively, and the absence of bands for ‐CHO (1676 cm^−1^) and C = N (1561 cm^−1^) from the building monomers, 2,5‐bis(2‐(2‐methoxyethoxy)ethoxy) terephthalaldehyde (BOPA) and 2,4,6‐trimethyl‐1,3,5‐triazine (TMT) (Figure 2d; Figure S2, Supporting Information). The ^13^C CP/MAS ssNMR spectra exhibited signals at ca. 178, 110‐116, and 50‐75 ppm, corresponding to the carbon atoms in triazine, vinylene, and alkoxy groups, respectively (Figure 2e; Figure S3, Supporting Information). The peak at 175 ppm may be attributed to attached TMT monomers.^[^ ^17^ ^]^ Furthermore, high‐resolution transmission electron microscopy (HRTEM) images (Figure 2f) revealed a periodic porous framework in all four COFs, with a π‐π stacking distance significantly stretching (from 0.31 to 0.39 nm) upon increasing the length of the ethoxy side chain, in agreement with the PXRD results. The phase purity of the COFs was further verified by scanning electron microscopy (SEM), which showed consistent morphologies for each COFs (Figure S4, Supporting Information).

In view of the harsh conditions of PEMWE application, it is important to measure the robustness of the synthesized COF scaffolds. Our COF‐O(n) were therefore subjected to immersion in 12 M HCl or 0.5 M H_2_SO_4_ for two days to assess acid resistance. Following this treatment, the FT‐IR spectra (Figure S5, Supporting Information) and PXRD patterns (Figure S6, Supporting Information) of protonated COF‐O(n) exhibited unchanged characteristic peaks, indicating that the atomic‐level connectivity and crystallinity were preserved. Additionally, the SEM images also showed that the COF morphologies remained intact (Figure S7, Supporting Information). The high degree of conjugation in the vinylene linkages provided COFs with remarkable acid stability, in agreement with previous reports.^[^ ^18^ ^]^ Thermogravimetric analysis (TGA) further confirmed their thermal stability within the operational temperature range, showing less than 1.5% weight loss up to 100 °C (Figure S8, Supporting Information).

Building on the robust structural stability of COFs in acidic conditions, hybrids of COFs and RuO_2_ (RuO_2_@COF‐O(n)) were prepared through vigorous sonication mixing. TEM images, combined with elemental mapping, revealed a uniform dispersion of RuO₂ and the COF structure within the RuO₂@COF‐O(n) hybrid (Figures S9‐S10, Supporting Information). High‐resolution Ru 3*p* X‐ray photoelectron spectroscopy (XPS) was performed, and the spectra were deconvoluted into two pairs of doublets. The Ru 3*p* spectra for the various catalysts displayed a primary doublet at 463.15 eV (3p_3/2_) and 485.34 eV (3p_1/2_), confirming the predominance of Ru^4+^ species in all samples. Additionally, a minor doublet at 466.08 eV and 488.56 eV was observed (Figure S11, Supporting Information), indicating the presence of satellites peaks.^[^ ^19^ ^]^ For the deconvoluted N 1*s* spectra, peaks at ≈398.4 eV and 399.9 eV correspond to nitrogen in triazine ring (N‐triazine) and pyrrolic nitrogen resulting from partial decomposition (Figure S12, Supporting Information), in line with previous reports.^[^ ^20^ ^]^ The noticeable negative shift in the Ru^4+^ peak and positive shift in the N‐triazine peak for RuO_2_@COF‐O(n) compared to pristine RuO_2_ and corresponding COF‐O(n) indicate a strong electron interaction between COFs and RuO_2_, confirming the effective integration of the catalysts. Additionally, the slight shifts in O 1*s* spectra suggest potential intermediate modification rather than electron rearrangement induced by the ethoxy side chains in COF‐O(n) (Figure S13, Supporting Information).

### 2.2 Structure‐Activity Relationship

**Figure 3:**
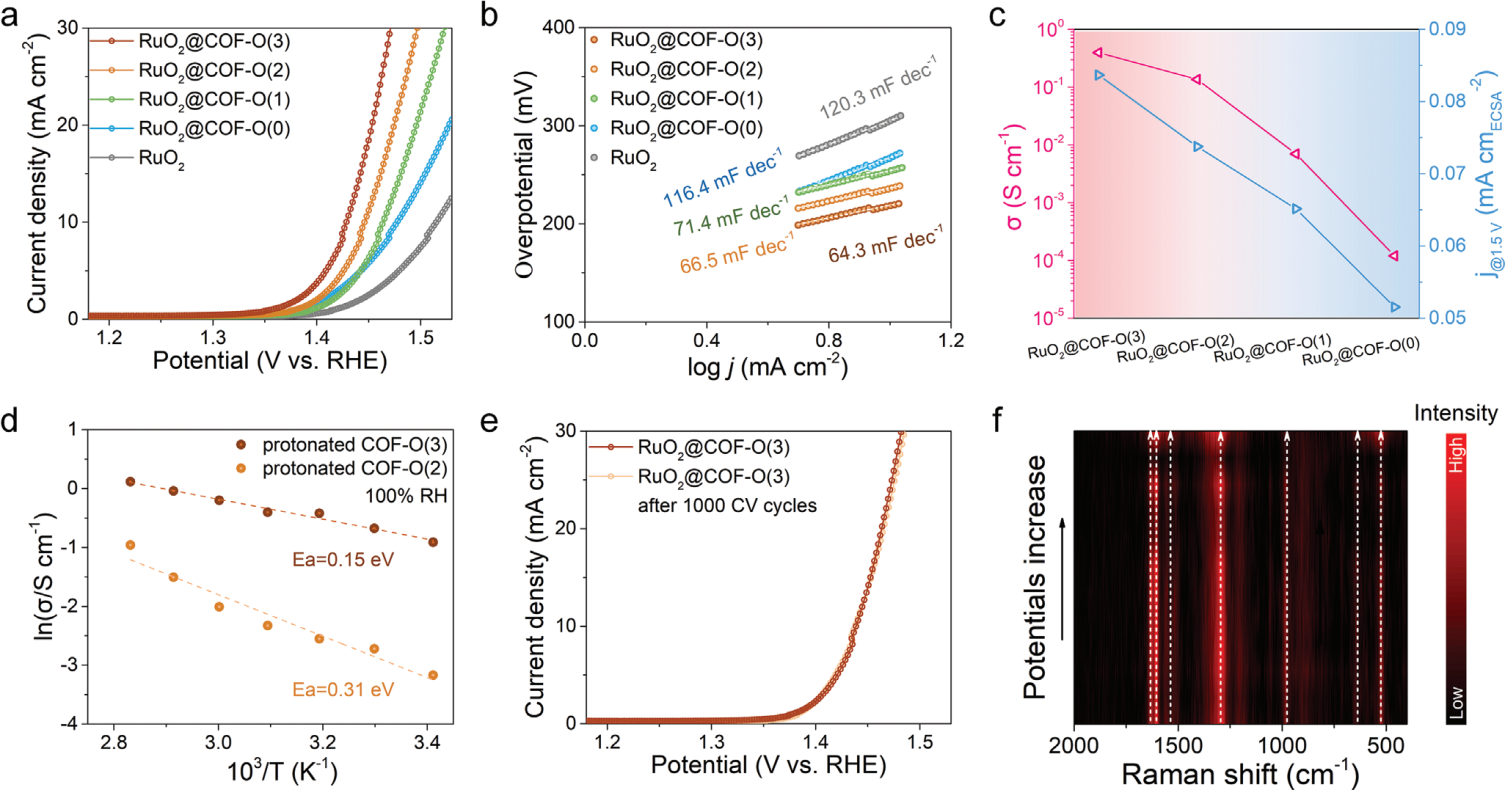
a) Polarization curves and b) Tafel slopes of RuO_2_@COF‐O(n), n = 0, 1, 2, and 3, and commercial RuO_2_. c) Relationship between proton conductivity (σ) at 80 °C and ECSA normalized current density at 1.5 V for RuO_2_@COF‐O(n), n = 0, 1, 2, and 3. d) Arrhenius plots of protonated COF‐O(3) and protonated COF‐O(2) under 100% relative humidity (RH). e) Polarization curves of RuO_2_@COF‐O(3) before and after 1000 cyclic voltammetry cycles. f) Raman spectra of RuO_2_@COF‐O(3) during OER tests, displaying several characteristic peaks corresponding to COF‐O(3) and RuO_2_, respectively.

The as‐prepared RuO_2_@COF‐O(n) hybrids were evaluated as acidic OER catalysts in a typical three‐electrode system with a 0.5 M H₂SO₄ electrolyte. We found that the overpotential at 10 mA cm^−2^ gradually decreased with the increasing length of ethoxy side chains, reaching the lowest value of 218 mV for RuO_2_@COF‐O(3) (**Figure** **3**a) followed by 236 mV, 255 mV, and 267 mV for RuO_2_@COF‐O(2), RuO_2_@COF‐O(1), and RuO_2_@COF‐O(0), respectively. All COF‐containing samples outperformed commercial RuO_2_, which had an overpotential of 305 mV. Taking into account the amount of active sites, electrochemically active surface area (ECSA) was measured (Figure S14, Supporting Information). RuO_2_@COF‐O(3) still exhibited the highest value of 30.2 cm_ECSA_^−2^, surpassing RuO_2_@COF‐O(2) (21.3 cm_ECSA_^−2^), RuO_2_@COF‐O(1) (15.8 cm_ECSA_^−2^), RuO_2_@COF‐O(0) (13.7 cm_ECSA_^−2^), and RuO_2_ (8.2 cm_ECSA_^−2^). This result indicates that upon increasing the length of the ethoxy side chains, the COF exposes more active sites. To further assess the intrinsic activity enhancement, linear sweep voltammetry (LSV) measurements were normalized with respect to ECSA, and the performance improvement relative to pristine RuO_2_ followed the trend: RuO_2_@COF‐O(3) > RuO_2_@COF‐O(2) > RuO_2_@COF‐O(1) > RuO_2_@COF‐O(0) (Figure S15, Supporting Information). The exceptional acidic OER activity of RuO_2_@COF‐O(3) was further confirmed by Tafel slope analysis, which showed a low value of 64.3 mV dec^−1^, significantly outperforming the other tested samples and indicating favorable kinetics during OER process (Figure 3b).^[^ ^21^ ^]^ Furthermore, our RuO_2_@COF‐O(3) material exhibits an exceptional acidic OER performance compared with most previously reported COF/MOF‐based electrocatalysts (Table S5, Supporting Information). Specifically, its combination of low overpotential, high current density, and robust stability underscores its potential as a benchmark material for future advancements in acidic OER catalysis.

To correlate the acidic OER performance of RuO₂@COF‐O(n) with their structural properties, proton conductivities of these functionalized COFs were estimated. The proton conductivities increased significantly from 0.12, 7.12, 135.66, to 396.82 mS cm⁻¹ for the protonated COF‐O(0), COF‐O(1), COF‐O(2), and COF‐O(3), respectively, demonstrating the effectiveness of extending the ethoxy chain length in enhancing proton conduction (Figure S16, Supporting Information). The high proton conductivity of COF‐O(3) is attributed to the hydrophilic oligoethylene glycol side chains, which facilitate efficient proton transport,^[^ ^22^ ^]^ and contribute to more oxygen sites for optimizing the spatial confinement of hydronium ions through electrostatic interactions.^[^ ^23^ ^]^ It was noteworthy that the trend in acidic OER performance closely matched the trend in the proton conductivity of the protonated COFs, indicating that the enhancement in performance is due to accelerated proton transfer (Figure 3c). Furthermore, the activation energy was estimated from the temperature dependence of proton conductivity. We found that protonated COF‐O(3) exhibits the lowest activation energy value of 0.15 eV, suggesting rapid proton transport via the Grotthuss mechanism (<0.4 eV) in water‐hydronium network (Figure 3d; Figure S17, Supporting Information).^[^ ^24^ ^]^ In addition, benefiting from the conjugated 2D skeleton with lateral conductive chains, RuO_2_@COF‐O(n) displayed lower charge transfer resistances than commercial RuO_2_, as evidenced by the smaller semicircles in electrochemical impedance spectroscopy (EIS) spectra (Figure S18 and Table S6, Supporting Information).^[^ ^25^ ^]^

In addition to catalytic activity, long‐term durability is a crucial factor for assessing the acidic OER performance of electrocatalysts. The LSV curves of RuO_2_@COF‐O(3) before and after 1000 cycles of cyclic voltammetry tests exhibited a negligible decay (Figure 3e). Additionally, the driving potential of RuO_2_@COF‐O(3) at a current density of 10 mA cm^−2^ increased slightly at an ultra‐low rate of ≈0.13 µv s^−1^ over 150 000 s, which was significantly more stable than that of RuO_2_ at the same current density (Figure S19, Supporting Information). The enhanced stability of RuO_2_@COF‐O(3) was further corroborated by inductively coupled plasma mass spectrometry (ICP‐MS), which revealed a significantly lower Ru leaching of 0.52 wt.% compared to 1.57 wt.% for commercial RuO_2_ after the stability test (Table S7, Supporting Information). This improved stability is attributed to the strong interaction between RuO₂ and the COF‐O(3) framework, which effectively mitigates Ru dissolution during acidic OER. The stability of the COF‐O(3) structure was confirmed by the unchanged characteristic peaks in the XRD pattern and FT‐IR spectrum after the OER stability test, demonstrating its high electrochemical stability under harsh conditions (Figures S20‐S21, Supporting Information).

To monitor the structural changes during acidic OER process, in situ electrochemical Raman spectroscopy was employed. The Raman peaks observed at ca. 983, 1292, 1552, 1604, and 1626 cm^−1^ were assigned to the C‐H, C‐O, C = N, C = C (phenyl), and C = C stretching bands, respectively, and were generally consistent with the simulated results, with only minor deviations due to different experimental conditions (Figure S22 and Table S8, Supporting Information). The peaks at 525 and 634 cm^−1^, corresponding to Ru‐O stretching bands, matched well with those of commercial RuO_2_ (Figure S23, Supporting Information). As the potential increased from open circuit voltage to 1.8 V versus reversible hydrogen electrode, the Raman shifts of the RuO_2_@COF‐O(3) remained stable, indicating the structural integrity of both protonated COF and RuO_2_ under oxidative reaction (Figure 3f and Figure S24, Supporting Information).^[^ ^26^ ^]^ In addition, the ab initio molecular dynamics simulations were performed under acidic conditions and applied potential, focusing on a segment of the COF that includes the triazine core and oligoethylene glycol lateral chain situated on the (110) plane of rutile RuO_2_ (Figure S25, Supporting Information). The three interaction, namely Ru‐O’, O‐C’, and O‐N’ between RuO_2_ and COF‐O(3) reached equilibrium after 1000 fs, with distances of 3.08, 3.28, and 3.90 Å, respectively. Combined with the robust COF‐O(3) structural model after simulation, COF‐O(3) with functional group exhibits high stability under applied potential and solvent model. Overall, our findings demonstrate the optimization of the length of ethoxy side chains in COFs effectively enhances proton conductivity, leading to notably improved OER activity and durability when hybridized with RuO_2_.

### 2.3 Mechanistic Study

In light of the pivotal role of COF‐O(3) in enhancing the stability of acidic OER, charge density distribution calculations were performed for RuO_2_@COF‐O(3) (Figure S26, Supporting Information). Both the COF's skeleton and the oligoethylene glycol chains exhibited strong interaction with the RuO₂ surface, resulting in efficient electron transfer from the COF structure to Ru sites, perfectly matching the XPS findings. The formation of electron‐rich Ru species is beneficial in preventing over‐oxidation into volatile RuO_4_ species, thus it enhances the stability of acidic OER.^[^ ^27^ ^]^

**Figure 4:**
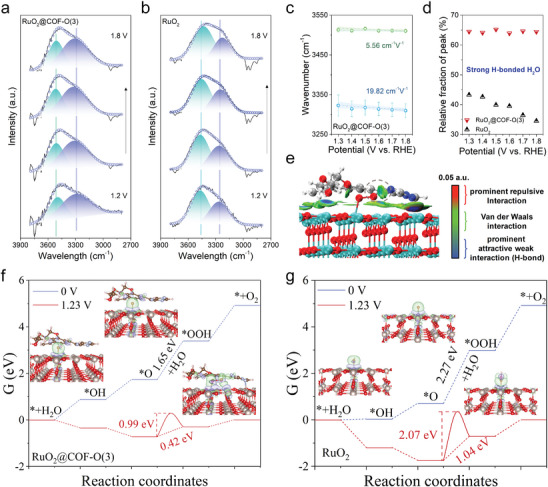
Part of in situ ATR‐FT‐IR spectra for a) RuO_2_@COF‐O(3) and b) RuO_2_. c) Stark tuning rate of RuO_2_@COF‐O(3). d) The variation in the fraction of the peak related to strong hydrogen bonding as the potential increased. e) Contour plots of independent gradient model based on Hirshfeld partition (IGMH) isosurfaces for RuO_2_@COF‐O(3) model adsorbed with *OOH intermediate, with blue surfaces indicating the hydrogen bonding and green surfaces indicating the van der Waals interactions. C, H, O, N, and Ru atoms are represented by grey, white, red, blue, and cyan. Free energy diagrams of reaction and kinetics barriers for f) RuO_2_@COF‐O(3) and g) RuO_2_ at applied potentials of 0 V and 1.23 V, illustrating the charge redistribution during intermediates adsorption at active sites for OER.

To gain deeper insights into the effects of COF‐O(3) in OER activity enhancement, in situ attenuated total reflectance infrared absorption spectroscopy (in situ ATR‐FT‐IR) was employed to investigate the interfacial water structures. The broad absorption feature observed between 3000 and 3610 cm^−1^ was attributed to O‐H stretching modes. This feature was further deconvoluted into two distinct peaks at ≈3480 cm^−1^ (highlighted in green) and ≈3270 cm^−1^ (highlighted in blue), corresponding to weakly and strongly hydrogen‐bonded interfacial water molecules, respectively (**Figure** **4**a‐b; Figure S27, Supporting Information).^[^ ^28^ ^]^ As positive potentials were gradually increased, RuO_2_@COF‐O(3) demonstrated a low Stark tuning rate and weak binding strengths, which are favorable for the deprotonation or desorption of intermediates (Figure 4c; Table S9, Supporting Information).^[^ ^29^ ^]^ In contrast, RuO_2_ exhibited a high Stark tuning rate, implying the stronger binding strengths that might restrict the formation of key oxo‐intermediate species (Figure S28 and Table S10, Supporting Information).^[^ ^30^ ^]^ As depicted in Figure 4d, pristine RuO_2_ experienced the potential‐dependent reorientation of water molecules, as evidenced by the shift from strongly hydrogen‐bonded vibrations in oxygen‐down orientation to weakly hydrogen‐bonded vibrations in oxygen‐up orientation as the potential increased.^[^ ^31^ ^]^ In contrast, RuO_2_@COF‐O(3) maintained a high proportion of strongly hydrogen‐bonded vibration with minimal reorientation of interfacial water molecules, underscoring the significant role of COFs in stabilizing the transition state for oxo‐intermediates formation. The persistent presence of oxygen‐down water molecules is particularly advantageous for deprotonation,^[^ ^32^ ^]^ thereby accelerating OER kinetics. Furthermore, the hydrogen bonds (shown in blue) and van der Waals interactions (shown in green) between COF‐O(3) and RuO_2_ adsorbed with key intermediates *OOH were clearly visualized,^[^ ^33^ ^]^ which also verified the contribution of hydrogen bonding in optimizing OER performance (Figure 4e; Figure S29, Supporting Information).

Given that the generation of key intermediates for acidic OER originated from the water dissociation step,^[^ ^34^ ^]^ the energy barriers for dissociating water molecules into *H and *OH species were calculated. In view of hydrogen‐bonded interaction with COFs, RuO_2_@COF‐O(3) facilitated water dissociation more effectively than RuO_2_, leading to a lower energy barrier of 0.809 eV compared with that of 1.338 eV for RuO_2_ (Figure S30, Supporting Information). Moreover, the pH‐dependent activity was examined to confirm the reaction pathway. As shown in Figure S31 (Supporting Information), both RuO_2_@COF‐O(3) and RuO_2_ displayed weak pH dependence, with reaction orders of ‐0.20 and ‐0.27, respectively, indicating a concerted proton‐electron transfer process in which proton transfer is involved in the RDS. To theoretically understand the catalytic mechanism, free energies of the four elementary steps (*→*OH→*O→*OOH→*O_2_) were further calculated. As shown in Figure 4f‐g, both RuO_2_@COF‐O(3) and RuO_2_ proceeded same catalytic pathway involving the adsorbate evolution mechanism, and the rate‐determining step was the formation of *OOH intermediate which exhibited the largest energy barriers of 1.65 and 2.27 eV, respectively. Furthermore, when comparing the kinetic energy barriers of the RDS, the RuO_2_@COF‐O(3) exhibits a significantly lower barrier of 0.99 eV, suggesting enhanced intermediate adsorption/desorption kinetics compared to RuO_2_, which has a higher barrier of 2.07 eV. The optimized OER performance was attributed to the strong coupling interaction between COFs and adsorbed intermediates, which accelerated proton transfer and deprotonation via a hydrogen‐bonded network (inset Figures of Figure 4f‐g).

### 2.4 Performance of PEMWE Devices

**Figure 5:**
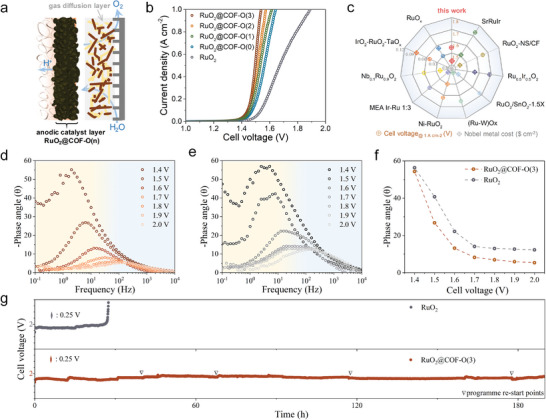
a) Schematic of the anodic composition layers and the reaction pathway in the PEMWE device. b) Polarization curves of the PEMWE electrolyzer obtained at 80 °C. c) Comparisons of the cell voltage required to achieve a 1.0 A cm^−2^ current density and anode noble metal cost for various RuO_2_‐based electrocatalysts in PEMWE. Bode phase plots of d) RuO_2_@COF‐O(3) and e) RuO_2_, respectively. f) Response of the phase angle to the applied potentials of RuO_2_@COF‐O(3) and RuO_2_. g) Chronopotentiometry curves at a current density of 200 mA cm^−2^.

For practical hydrogen production applications, it is crucial to assess the performance of the PEMWE setup within a more complex reaction environment. A series of RuO_2_@COF‐O(n) hybrids were used as anodic catalyst layer and then were uniformly coated onto the surface of Nafion 117 membranes, as confirmed by energy‐dispersive X‐ray spectroscopy (Figure S32, Supporting Information). The robust mechanical properties of the catalyst‐coated membrane (CCM) were evidenced by its ability to maintain a flat surface even after undergoing 50 bending cycles (Figure S33, Supporting Information). The catalyst layers’ thicknesses were kept constant across samples to minimize performance variability (Figure S34, Supporting Information). The assembled PEMWE devices were tested at 80 °C with distilled water supplied to the anode side (**Figure** **5**a; Figure S35, Supporting Information). The RuO_2_@COF‐O(3) electrolyzer achieved a high current density of 1.0 A cm^−2^ with a low voltage of only 1.54 V, significantly outperforming commercial RuO_2_ at 1.89 V (Figure 5b). This performance enhancement was notably greater than that observed in the three‐electrode system, highlighting the crucial role of COFs under PEMWE conditions. Such low voltage resulted in an estimated energy consumption of just 41 kWh per kilogram of H_2_ (Note S1, Supporting Information).^[^ ^26^, ^35^ ^]^ Specifically, combining COF‐O(3) with RuO_2_ not only effectively exposed more active sites but also achieved a low noble metal cost (0.011 $ cm^−2^) with low loading amount of 0.76 mg_Ru_ cm^−2^. Furthermore, the exceptional performance of RuO_2_@COF‐O(3) surpassed most previously reported RuO_2_‐based OER electrocatalysts (Figure 5c; Table S11, Supporting Information).

To further elucidate the impact of functionalized COF in the PEMWE device, in situ EIS measurements were conducted. At low voltages, the charge transfer resistance for both RuO_2_@COF‐O(3) and RuO_2_ was primarily influenced by the electrolyte‐catalyst interface, while at high voltages it was determined by intrinsic electron conduction. This was evidenced by the shifting of phase peaks from low‐frequency regions (0.01–10 Hz) to high‐frequency regions (100–10000 Hz) in the Bode phase plots as the applied voltage increased (Figure 5d‐e).^[^ ^36^ ^]^ It is evident that the phase angles for RuO_2_@COF‐O(3) decreased more rapidly than those for RuO_2_ throughout the entire reaction process (Figure 5f), indicating that combining RuO₂ with functionalized COFs enhances OER reaction kinetics. In addition to its high activity, RuO_2_@COF‐O(3) demonstrated a stable PEMWE performance over 180 h at a constant industrial‐level current density of 200 mA cm^−2^, underscoring the practical application potential of the functionalized COFs (Figure 5g). Notably, the surface composition of the RuO_2_@COF‐O(3) CCM after extended stability testing demonstrated excellent retention of the hybrid structure. The PXRD patterns and Raman spectra exhibited well‐preserved characteristic peaks of COF‐O(3) and RuO₂, while the elemental mapping showed a uniform distribution of Ru, C, N, and O across the surface (Figures S36–S37, Supporting Information). These observations confirm the structural and compositional stability of the RuO₂@COF‐O(3) CCM during long‐term PEMWE operation.

## 3 Conclusion

In summary, we developed functional COFs possessing high acid resistance and proton conductivity to serve as an active adlayer at catalyst‐water interface. The COFs’ conjugated skeleton and lateral proton conductive chains enhanced their interaction with RuO₂, facilitating rapid proton hopping. Combined insitu spectroscopic, electrochemical experiments and theoretical analyses revealed that the exceptional acidic OER performance of RuO_2_@COF‐O(3) stemmed from the stabilization of transition states for oxo‐intermediates adsorption and desorption, mediated by COF‐defined oriented interfacial water molecules. The optimized PEMWE setup achieved a low cell voltage of only 1.54 V at 1.0 A cm^−2^ and low noble metal cost of 0.011 $ cm^−2^, with the estimated energy consumption of 41 kWh per kg_H2_. This oriented hydrogen‐bonded network underscores the critical role of hydrogen bonding oxo‐intermediate transformation and demonstrates how functionalized COFs can advance PEMWE technology.

## 4 Experimental Section

### Synthesis of COF‐O(3)

A mixture of 5.0 mg 2,4,6‐trimethyl‐1,3,5‐triazine, 22.56 mg 2,5‐Bis(2‐(2‐methoxyethoxy)ethoxy) terephthalaldehyde (BOPA), 13.78 mg benzoic anhydride, and 0.74 mg benzoic acid was sealed into a Pyrex tube under vacuum after being degassed by three freeze‐pump‐thaw cycles at 77 K (liquid N_2_). The tube was heated at 180 °C for 5 days. After cooling to room temperature, the precipitated powder was ground with a mortar and washed three times each with tetrahydrofuran, ammonia, and acetone. The sample was then dried under vacuum at 100 °C overnight.

### Characterizations

Powder X‐ray diffraction (PXRD, BrukerD8 X‐ray diffractometer) was tested to characterize the crystal structure of as‐prepared samples. Field‐emission scanning electron microscope (FESEM, FEI Quanta FEG 250 instrument) and high‐resolution transmission electron microscope (HRTEM Tecnai G2 F20S‐TWIN) were applied to observe the microscopic morphology of as‐prepared samples. Energy‐dispersive X‐ray spectroscopy (EDX, Oxford X‐max80) equipped with FESEM was conducted to verify the actual distribution of elements in samples. X‐ray photoelectron spectroscopy (XPS, Thermo Scientific, Escalab 250Xi) with Al Kα radiation was utilized to analyze the valence of major elements in samples. All XPS spectra were calibrated using the C 1s peak at 284.8 eV as a reference. Raman spectroscopy was collected by a Renishaw inVia spectrometer equipped with 780 nm laser. Fourier transform infrared (FT‐IR) tests were carried out by A BRUKER INVENIO R spectrometer equipped with a liquid nitrogen‐cooled MCT detector. The ^13^C cross‐polarization/magic angle spinning solid‐state nuclear magnetic resonance (^13^C CP/MAS ssNMR) spectra were recorded on a Bruker Avance ll HD spectrometer coupled to an 11.7 T wide bore superconducting magnet operating at 125.76 MHz ^13^C Larmor frequency.

### Electrochemical Tests

Electrochemical OER measurement was employed at room temperature using an electrochemical analyzer (PGSTAT204) in a typical three‐electrode configuration where the graphite rod, glassy carbon, and Ag/AgCl electrode worked as counter electrode, working electrode, and reference electrode, respectively. The glassy carbon electrode was first polished to a mirror‐like finish using a series of alumina slurries with particle sizes from 5 to 0.05 µm. To generate a homogeneous ink, 10.0 mg of catalyst was added to 1.0 mL of ethanol containing 10 µL Nafion aqueous solution, and dispersed by sonication for 30 min. 2.0 µL of the catalyst ink was drop‐casted on the surface of the working electrode with a loading amount of 0.28 mg_RuO2_ cm^−2^ and dried in air at room temperature. Before testing, the electrocatalyst was cycled in the potential ranges from 0.9 to 1.4 V versus Ag/AgCl until a steady cyclic voltammetry curve was obtained. LSV was tested at a scan rate of 5 mV s^−1^. The ECSA was measured from the electrochemical double‐layer capacitance (C_d1_) by analyzing cyclic voltammetry curves at various scan rates of 10, 30, 50, 70, 90, and 110 mV s^−1^. The frequency of EIS was arranged from 100 kHz to 0.01 Hz and the EIS results were presented in the form of Nyquist plot. All the measured potentials in 0.5 M H_2_SO_4_ electrolyte were converted to the reversible hydrogen electrode (RHE) via the Nernst equation (E_RHE_ = E_Ag/AgCl_ + 0.059 × pH + 0.197). All polarization curves were calibrated with iR‐correction with the following equation: E_final_ = E_0_ – (iR) V, in which E_0_ is the potential referenced to the polarization curve, i is the current at E_0_ and R results from the EIS figures.

### PEMWE Tests

A home‐made proton exchange membrane water electrolysis (PEMWE device was used for single‐cell measurements, which was composited by platinum‐coated titanium meshes as gas diffusion layers, titanium plates as two electrodes, and catalysts‐coated membrane (CCM) as reaction zone. The sandwich structure of CCM was fabricated by a spray coater to uniformly coat the catalyst inks on the PEEK substrates followed by hot‐pressing onto the PEM (Nafion 117, 175 µm) at 120 °C and 0.5 MPa for 180 s. The anodic catalyst ink consisted of 15.0 mg of commercial RuO_2_, 4.0 mg functionalized COFs, and 120 mg of Nafion solution was dissolved in 12 mL isopropanol and intermittently ultrasonicated for 24 hours. The mass loading of Ru in CCM was maintained as 0.76 mg_Ru_ cm^−2^. The cathodic catalyst ink consisted of 30.0 mg of commercial Pt/C and 288 mg of Nafion solution was dissolved in 12 mL isopropanol and ultrasonicated for 10 hours. The mass loading of Pt in CCM was maintained as 0.07 mg_Pt_ cm^−2^ for all the tests. The electrochemical measurements were operated at a temperature of 80 °C using distilled water as the electrolyte under the flowing rate of 13  mL min^−1^. The performance of the PEMWE device was evaluated using an electrochemical analyzer (PGSTAT128N). All polarization curves were measured from 1.0 to 2.5 V and corrected based on iR‐correlation.

### DFT Calculations

The first principles calculations were used by Vienna ab initio Simulation Package.^[^ ^37^ ^]^ A plane‐wave cutoff energy of 450 eV was applied. Generalized gradient approximation proposed by Perdew, Burke, and Ernzerh with was used in the projector augmented wave method.^[^ ^38^ ^]^ To accurately capture weak interactions, the computationally efficient Grimme's D3 scheme method for van der Waals (vdW) interactions was employed.^[^ ^39^ ^]^ A vacuum separation of 15 Å between neighboring RuO_2_ slabs was maintained due to periodic boundary conditions. Brillouin zone sampling was performed using the Monkhorst‐Pack scheme.^[^ ^40^ ^]^ Ionic and electronic relaxations were carried out with convergence criterion set at 0.05 eV/Å per ion and 10^−5^ eV per electronic step, respectively. Ab initio molecular dynamics simulation was performed under a constant potential using the CP‐DFT method, and the target potential set to 1.23 V.^[^ ^41^ ^]^ The Gibbs free energies (G) of each reaction intermediates were calculated using G = E_DFT_ + E_ZPE_ – TS, where E_DFT_ represents total energy from DFT calculations, E_ZPE_ denotes zero‐point energy, T, and S were temperature (300 K) and entropy, respectively. The climbing‐image nudged elastic band method was used for the decomposition barriers of H_2_O.^[^ ^42^ ^]^

## Conflict of Interest

The authors declare no conflict of interest.

## Supporting information



Supporting Information

## Data Availability

The data that support the findings of this study are available from the corresponding author upon reasonable request.
