# Supplementary material for: Supramolecular Engineering of Vinylene‐Linked Covalent Organic Framework – Ruthenium Oxide Hybrids for Highly Active Proton Exchange Membrane Water Electrolysis
Source: Adv Mater. 2025 Feb 3;37(11):2417374. doi: 10.1002/adma.202417374 (PMC11923516; doi:10.1002/adma.202417374)
Supplement: Supplementary file 1 — Supporting Information [file ADMA-37-2417374-s001.docx]

Supporting Information

**Supramolecular engineering of covalent organic framework - ruthenium oxide hybrids for highly active proton exchange membrane water electrolysis**

*Kexin Wang, Shunqi Xu, Dashuai Wang, Zhenhui Kou, Yubin Fu, Michał Bielejewski, Verónica Montes-García, Bin Han, Artur Ciesielski,* Yang Hou,* and Paolo Samorì**

**Methods**

**Materials**

2,4,6-trimethyl-1,3,5-triazine (TMT), terephthalaldehyde (TPDA), 2,5-dimethoxy terephthalaldehyde (DMTA), 2,5-bis(2-methoxyethoxy)ethoxy terephthalaldehyde (BOEA), 2,5-dihydroxyterephthalaldehyde, 1-bromo-2-(2-methoxyethoxy)ethane, benzoic anhydride, benzoic, and ethyl acetate were obtained from BLD pharm. Propan-2-ol, tetrahydrofuran (THF), *N, N*-dimethylformamide, and dichloromethane (DCM) were obtained from Fishel Scientific. Ethanol absolute anhydrous (CARLO ERBA Reagents), RuO_2_ (Alfa Aesar), Pt/C (10 wt.% Sigma-Aldrin), Nafion solution (5 wt.%, Sigma-Aldrich), and potassium carbonate (Sigma-Aldrich) were bought. All the chemicals were used as obtained without further purification.

**Synthesis of BOPA**

The 2,5-Bis(2-(2-methoxyethoxy)ethoxy) terephthalaldehyde (BOPA) was synthesized according to reported literature with a modified procedure.^[1]^ 2,5-dihydroxyterephthalaldehyde (0.3 g, 1.81 mmol), 1-bromo-2-(2-methoxyethoxy)ethane (0.99 g, 5.42 mmol), and potassium carbonate (0.54 g, 3.79 mmol) were added into a degassed *N, N*-dimethylformamide (20 mL) under N_2_ protected atmosphere. Then the mixture was refluxed overnight. After cooling down, the mixture was poured into brine (100 mL) and extracted with dichloromethane (DCM) (30 mL) for 3 times. The combined organic layers were dried with Na_2_SO_4_, and then evaporated. Afterward, the mixture was purified by column chromatography over silica gel (DCM / ethyl acetate = 50 : 1 ~2 : 1) to give BOPA as green-yellow powder.

**Synthesis of COF-O(2).**

A mixture of 7 mg 2,4,6-trimethyl-1,3,5-triazine (TMT), 24.07 mg 2,5-bis(2-methoxyethoxy)ethoxy terephthalaldehyde (BOEA), 19.29 mg benzoic anhydride, and 1.04 mg benzoic acid was sealed into a Pyrex tube under vacuum after being degassed by three freeze-pump-thaw cycles at 77 K (liquid N_2_). The tube was heated at 180 ^o^C for 5 days. After cooling to room temperature, the precipitated powder was ground with a mortar and washed three times each with tetrahydrofuran (THF), ammonia, and acetone. The sample was then dried under vacuum at 100 °C overnight.

**Synthesis of COF-O(1).**

A mixture of 10 mg TMT, 23.65 mg 2,5-dimethoxy terephthalaldehyde (DMTA), 55.11 mg benzoic anhydride, and 29.75 mg benzoic acid was sealed into a Pyrex tube under vacuum after being degassed by three freeze-pump-thaw cycles at 77 K (liquid N_2_). The tube was heated at 180 ^o^C for 5 days. After cooling to room temperature, the precipitated powder was ground with a mortar and washed three times each with THF, ammonia, and acetone. The sample was then dried under vacuum at 100 °C overnight.

**Synthesis of COF-O(0).**

A mixture of 10 mg TMT, 16.34 mg terephthalaldehyde (TPDA), 55.11 mg benzoic anhydride, and 29.75 mg benzoic acid was sealed into a Pyrex tube under vacuum after being degassed by three freeze-pump-thaw cycles at 77 K (liquid N_2_). The tube was heated at 180 ^o^C for 5 days. After cooling to room temperature, the precipitated powder was ground with a mortar and washed three times each with THF, ammonia, and acetone. The sample was then dried under vacuum at 100 °C overnight.

**Synthesis of protonated COF-O(n).**

In order to accurately confirm the structure and property of COF-O(n) under acidic working conditions, the protonated COF-O(n) (n=0, 1, 2, and 3) samples were prepared by immersing COF-O(n) in 0.5 M H_2_SO_4_ for 24 hours, followed by filtration with distilled water and ethanol, and drying at 100 ^o^C overnight. These samples were then used for proton conductivity tests and XPS measurements.

**Electrochemical active surface area calculations**

The electrochemical active surface area (ECSA) of samples was determined using the following equation:

Eq1


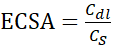

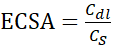


Where C_dl_ is the electrical double-layer capacitor which were measured from double-layer charging curves using cyclic voltammograms in a non-Faradaic region, and C_S_ is the specific capacitance of ideal metal oxides with smooth surface (0.06 mF cm^-2^).

**Proton conductivity measurements.**

For the electrochemical impedance spectroscopy (EIS) measurements, a series of protonated COF-O(n) rectangle pieces were prepared by applying a pressure of 10 MPa for 4 minutes, yielding an electrode with 0.3 × 0.3 cm^2^ and 0.1 cm thickness in size. The in-plane proton conductivity of the thin block was measured with two gold electrodes. The whole cell assembly was maintained in a humidity chamber to control the temperature and humidity. EIS was measured on an electrochemical workstation (PGSTAT204) with an amplitude of 5 mV in the frequency range of 1 MHz to 100 Hz.

Proton conductivity (
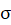
) calculation equation:


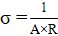
 Eq 2

where l is the thickness of the piece, A is the electrode cross-sectional area, and R is the proton resistance of the material.

Activation energy (Ea) calculation equation:


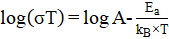
 Eq 3

where σ is the conductivity, k_B_ is the Boltzmann constant, A is the pre-exponential factor, and T is the temperature.

The effect of temperature on protonated COF-O(n) was investigated by keeping the relative humidity (RH) constant at 100 % and varying the temperature between 20 °C and 80 °C. The proton resistance was obtained from the fitted semicircular arc in the Nyquist plot. Typically, if the sample has high conductivity so that the semicircular arc is not clear, the intercept with the x-axis is considered the value of resistance.

**In-situ ATR-FTIR experiments.**

The BRUKER INVENIO R spectrometer equipped with a liquid nitrogen-cooled MCT detector was used for in-situ ATR-FTIR measurements. A silicon facet crystal with an incident angle of 60° was applied as reflective element. The ultra-thin gold foil was chemically deposited on the silicon to enhance in-situ ATR-FTIR signal and electronic conduction. In all tests, Ag/AgCl and Pt electrodes worked as counter electrode and reference electrode, respectively and the electrolyte was 0.5 M H_2_SO_4_ solution. In this experiment, chronopotentiometry test was tested at different potentials without in-situ ATR-FTIR correction. The potential was set from 1.2 to 1.8 V, and the electric potential of the reference single beam spectrum was 0.1 V.

**Raman simulation**

Geometrical optimization of the COF structure was carried out using density functional theory (DFT) at the M062X/6-311++G** level, followed by analysis of their vibrational frequencies to characterize the structures obtained as energy minima. These computations were performed using Gaussian16. The description of the vibrational modes and their assignment to the theoretical Raman spectrum was performed by visual inspection of the atomic displacements for each vibrational mode.

**Independent gradient model**

In order to analysis the weak interactions between the layers in the molecules, independent gradient model based on Hirshfeld partition (IGMH) method in Multiwfn was performed.^[2]^ To obtain the wavefunction for analysis, single-point energy calculation was performed by using the CP2K program.^[3]^ The PBE functional with the DZVP-MOLOPT-SR-GTH basis set were used. The convergence threshold for all self-consistent field (SCF) calculations is set to be 1.0 × 10^−6^ Hartree. To get better accuracy, we used Grimme’s D3 van der Waals dispersion correction.^[4]^ The isosurface maps were rendered by VMD program.^[5]^

**Figure S1.** PXRD patterns of a) COF-O(2), b) COF-O(1), and c) COF-O(0): Pawley refined pattern (black), experimental pattern (red), differences (green), simulated eclipsed (AA) stacking pattern (blue), and simulated eclipsed (AB) stacking pattern (pink).

**Figure S2.** FT-IR spectra of a) COF-O(2), b) COF-O(1), c) COF-O(0), and corresponding BOEA, DMTA, TPTA, and TMT monomers.

**Figure S3.** ^13^C CP/MAS NMR spectra of a) BOPA, b) COF-O(2), c) COF-O(1), and d) COF-O(0).


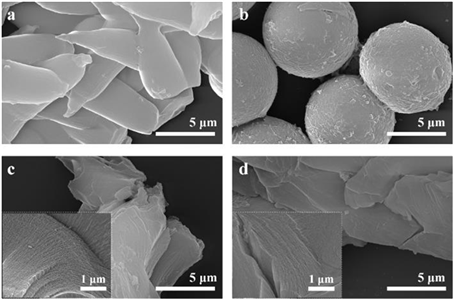


**Figure S4.** a-d) SEM images of COF-O(n), n = 0, 1, 2, and 3.

**Figure S5.** FT-IR spectra of a) COF-O(3), b) COF-O(2), c) COF-O(1), d) COF-O(0) before and after protonation treatments in 0.5 M H_2_SO_4_.

**Figure S6.** XRD patterns of a) COF-O(3), b) COF-O(2), c) COF-O(1), d) COF-O(0) before and after protonation treatments.


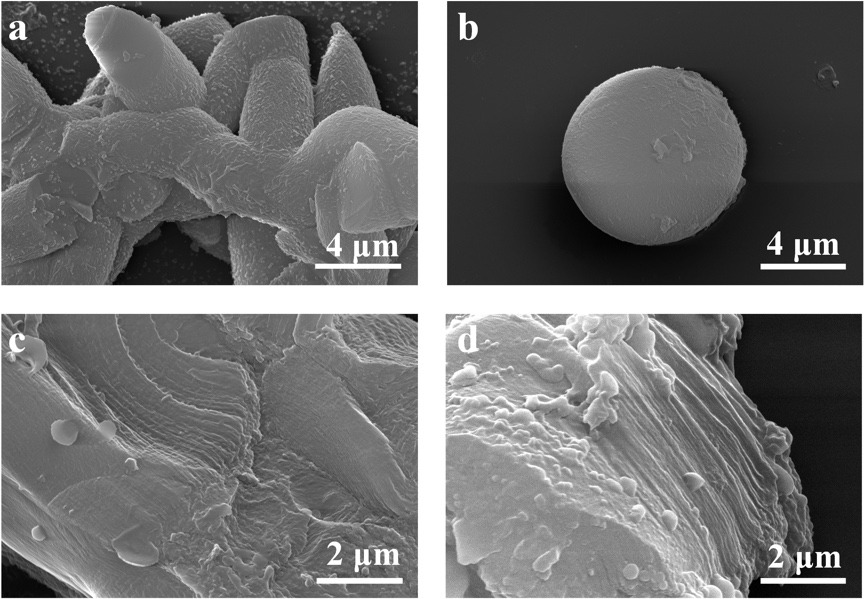


**Figure S7.** a-d) SEM images of COF-O(n), n = 0, 1, 2, and 3 after protonation treatment in 0.5 M H_2_SO_4_.

**Figure S8.** TGA curves for COF-O(n), n = 0, 1, 2, and 3.


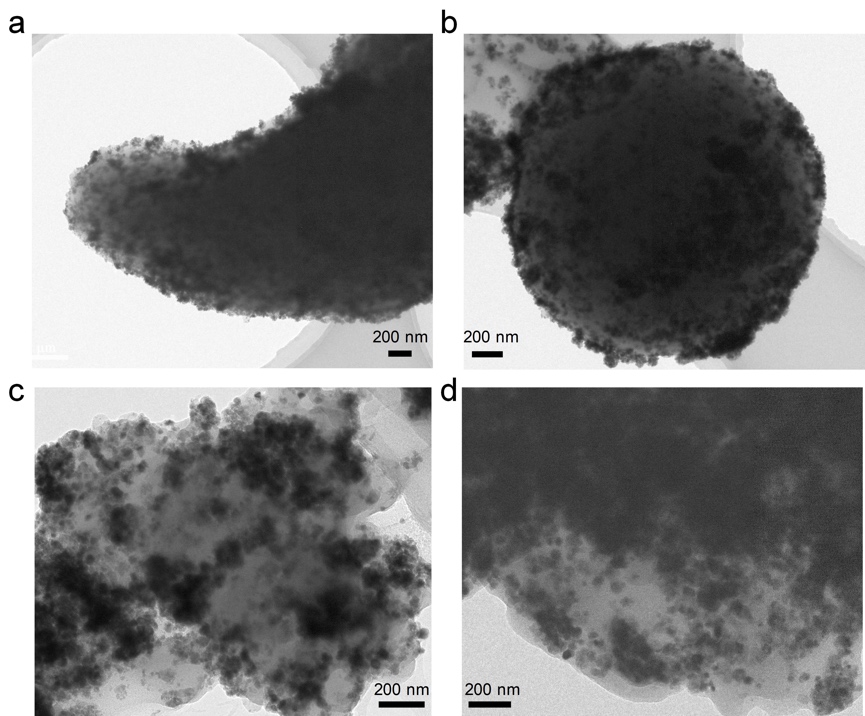


**Figure S9.** a-d) TEM images of RuO_2_@COF-O(n), n = 0, 1, 2, and 3.


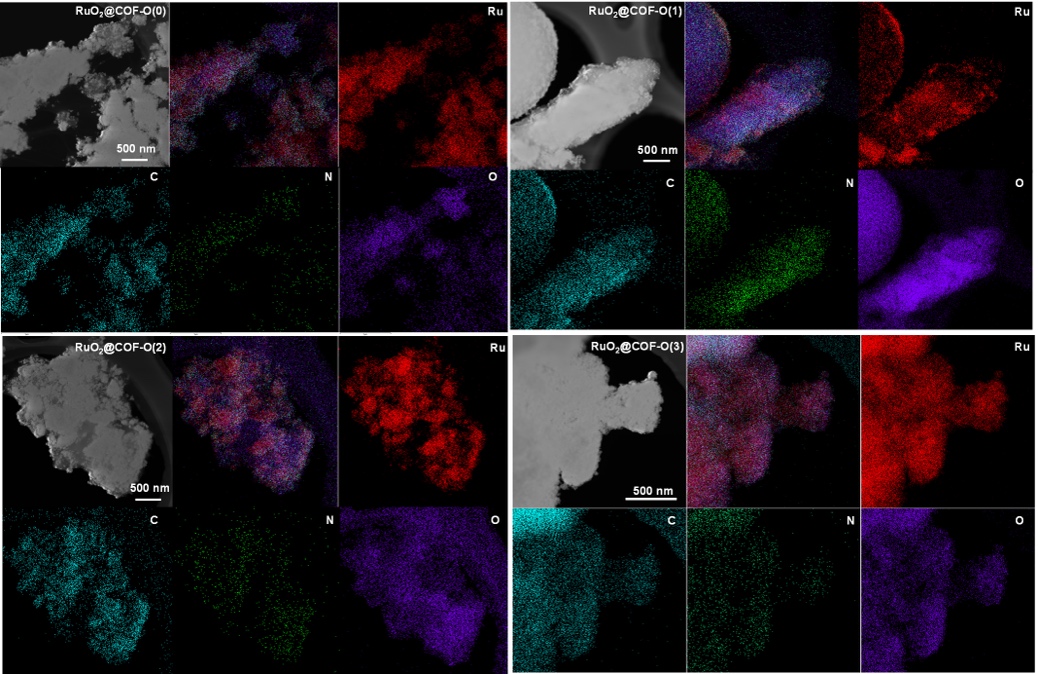


**Figure S10.** a-d) TEM-EDX mapping images of RuO_2_@COF-O(n), n = 0, 1, 2, and 3.

**Figure S11.** The high-resolution Ru 3p XPS spectra of RuO_2_@COF-O(n), n=0, 1, 2, and 3, and RuO_2_, where COF were previously protonated to mimic the acidic operating environment.

**Figure S12**. The high-resolution N 1*s* XPS spectra of RuO_2_@COF-O(n), n=0, 1, 2, and 3, and corresponding pristine COF-O(n).

**Figure S13**. The high-resolution O 1*s* XPS spectra of RuO_2_@COF-O(n), n=1, 2, and 3, and pristine RuO_2_ as well as corresponding COF-O(n).

**Figure S14.** a) C_dl_ diagram of RuO_2_@COF-O(3), RuO_2_@COF-O(2), RuO_2_@COF-O(1), RuO_2_@COF-O(0), and RuO_2_, and b-f) corresponding CV curves at different scan rates (mV s^-1^).

**Figure S15.** LSV curves of OER normalized by ECSA surface area for RuO_2_@COF-O(3), RuO_2_@COF-O(2), RuO_2_@COF-O(1), RuO_2_@COF-O(0), and RuO_2_.

**Figure S16.** a-c) Proton conductivity (σ) of protonated COF-O(n), n=0, 1, 2, and 3.

**Figure S17.** Arrhenius plots of protonated COF-O(1) and protonated COF-O(0) under 100 % RH.

**Figure S18**. Nyquist plots of RuO_2_@COF-O(3), RuO_2_@COF-O(2), RuO_2_@COF-O(1), RuO_2_@COF-O(0), and RuO_2_.

**Figure S19.** Chronopotentiometry curves of RuO_2_@COF-O(3) and RuO_2_ (glassy carbon electrode) at a current density of 10 mA cm^−2^.

**Figure S20.** XRD patterns of COF-O(3) before and after stability tests.

**Figure S21.** FT-IR spectra of RuO_2_@COF-O(3) before and after stability tests.

**Figure S22.** Experimental and simulated Raman spectra of COF-O(3).

**Figure S23.** a) Comparison of Raman spectra for RuO_2_@COF-O(3), COF-O(3), and RuO_2_.

**Figure S24.** a) In situ Raman spectra of RuO_2_@COF-O(3) from open circuit voltage (OCV) to 1.8 V. b) Diagram of peak shifts at 525 and 634 cm^-1^ for Ru-O stretching bands.

**Figure S25**. a) The constructed model of RuO_2_@COF-O(3) hybrid for AIMD simulation from the perspectives of top and front views. b) The simulated atomic distances for Ru-O’, O-C’, and O-N’ interactions between RuO_2_ and COF-O(3).

**Figure S26.** Charge density difference between RuO_2_@COF-O(3) and RuO_2_ from the perspectives of a) the top view and b) the front view.

**Figure S27.** Part of in situ ATR-FT-IR spectra for (a) RuO_2_@COF-O(3) and (b) RuO_2_.

**Figure S28.** Stark tuning rate of RuO_2_.

**Figure S29.** Contour plots of independent gradient model based on Hirshfeld partition (IGMH) iso-surfaces for RuO_2_@COF-O(3) model adsorbed with *OOH intermediate, with blue surfaces indicating the hydrogen bonding and green surfaces indicating the noncovalent interactions. C, H, O, N, and Ru atoms are represented by grey, white, red, blue, and cyan, respectively.

**Figure S30.** Energy barrier diagram of the water dissociation for RuO_2_@COF-O(3) and RuO_2_ from initial state (IS) to final state (FS).

**Figure S31**. LSV curves of RuO_2_@COF-O(3) and RuO_2_ under H_2_SO_4_ electrolytes with different pH values.


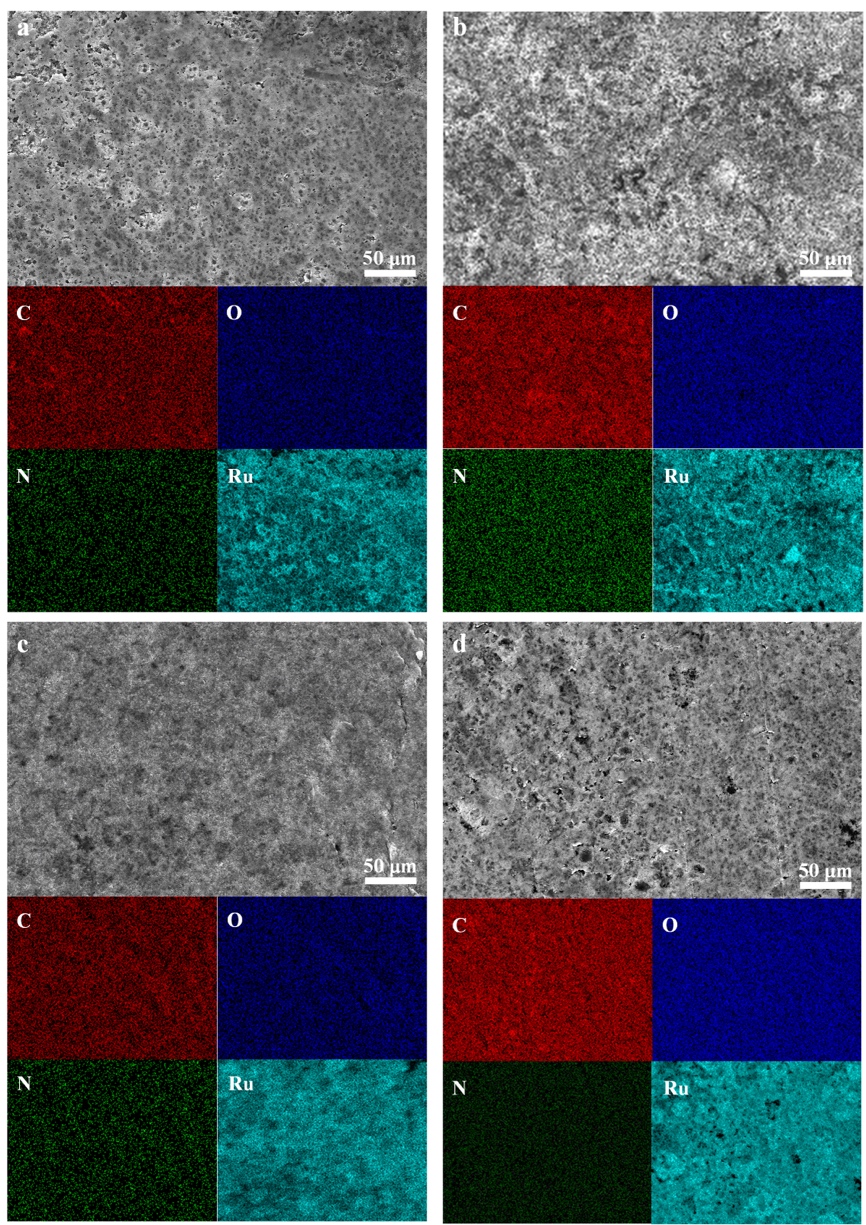


**Figure S32.** a-d) FESEM and corresponding EDX elemental mapping images of the surface of RuO_2_@COF-O(n) (n = 0, 1, 2, and 3) coated membranes, including the C, O, N, and Ru elements.


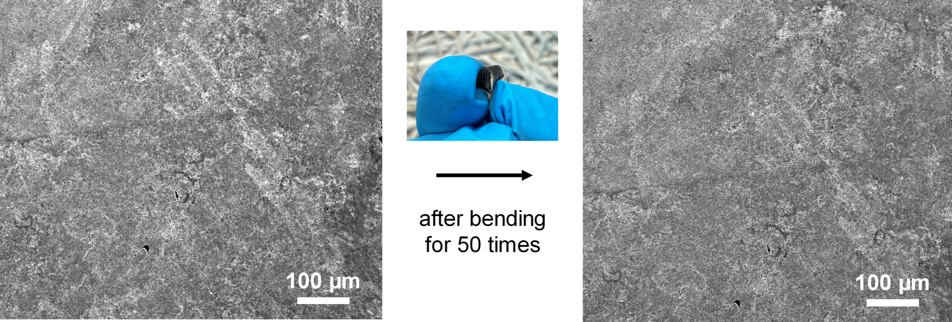


Figure S33. SEM images of CCM surface before and after 50 cycles of bending.


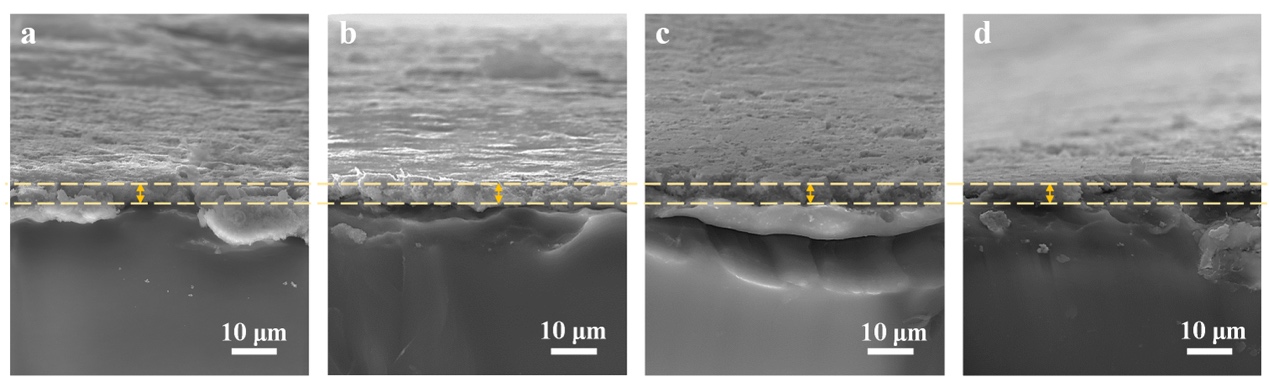


**Figure S34.** a-d) Cross-sectional images of the anodic catalyst layer for RuO_2_@COF-O(n) (n = 0, 1, 2, and 3) coated membranes, showing an average thickness of ~ 4.5 μm.


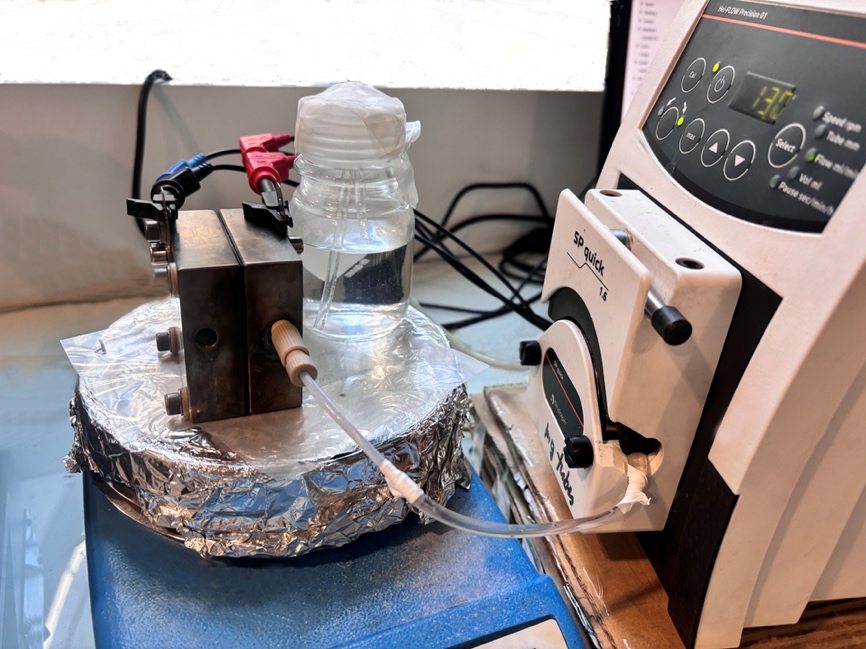


**Figure S35.** Photograph of PEMWE device.

Figure S36. a) XRD patterns and Raman spectra of RuO_2_@COF-O(3) CCM after long-term stability test.


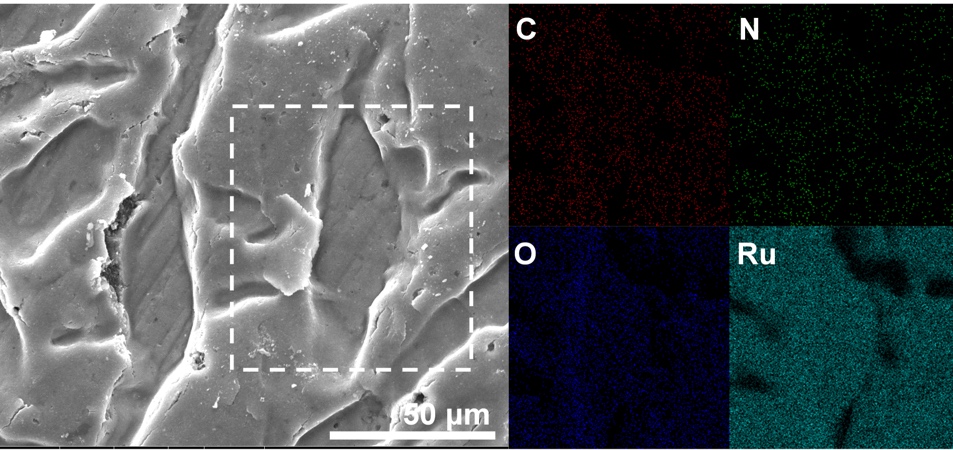


Figure S37. FESEM and corresponding EDX elemental mapping images of the surface of RuO_2_@COF-O(3) CCM after long-term stability test, including the C, O, N, and Ru elements.

**Table S1.** Fractional atomic coordinates for the eclipsed (AA) stacking unit cell of COF-O(3).

Crystal system: Hexagonal

Space group: P1

International tables number: 1

a= 21.4704 Å, b= 21.5769 Å, c= 4.5824 Å

α= β= 90^o^, γ= 116.47^o^

| Atom | x/a | y/b | z/c |
| --- | --- | --- | --- |
| N1 | 0.58324 | -0.70538 | -0.23508 |
| C2 | 0.60687 | -0.75258 | -0.22804 |
| N3 | 0.66852 | -0.73989 | -0.11149 |
| C4 | 0.70134 | -0.68019 | 0.03485 |
| N5 | 0.67633 | -0.63443 | 0.05971 |
| C6 | 0.61725 | -0.64897 | -0.07731 |
| C7 | 0.77426 | -0.658 | 0.14104 |
| C8 | 0.81398 | -0.59261 | 0.24362 |
| C9 | 0.89169 | -0.55898 | 0.2977 |
| C10 | 0.92714 | -0.59917 | 0.27715 |
| C11 | 0.99892 | -0.57209 | 0.27005 |
| C12 | 1.03684 | -0.49926 | 0.27913 |
| C13 | 1.00266 | -0.45907 | 0.328 |
| C14 | 0.931 | -0.4868 | 0.34072 |
| C15 | 1.1128 | -0.46313 | 0.22379 |
| C16 | 1.16105 | -0.41319 | 0.38967 |
| C17 | 1.23484 | -0.39729 | 0.34165 |
| N18 | 1.24745 | -0.45048 | 0.27344 |
| C19 | 1.31125 | -0.43476 | 0.18449 |
| N20 | 1.36145 | -0.37043 | 0.16111 |
| C21 | 1.34462 | -0.32078 | 0.23787 |
| N22 | 1.28074 | -0.3318 | 0.3147 |
| C23 | 1.394 | -0.24924 | 0.15958 |
| C24 | 1.32786 | -0.49225 | 0.10279 |
| C25 | 1.37251 | -0.21151 | -0.01148 |
| C26 | 1.37766 | -0.48249 | -0.09643 |
| C27 | 1.41147 | -0.52908 | -0.13133 |
| C28 | 1.41665 | -0.13873 | -0.10393 |
| C29 | 1.3722 | -0.59547 | -0.23829 |
| C30 | 1.39982 | -0.64187 | -0.27661 |
| C31 | 1.47066 | -0.61953 | -0.20973 |
| C32 | 1.50871 | -0.5541 | -0.09007 |
| C33 | 1.48123 | -0.5079 | -0.04738 |
| C34 | 1.48562 | -0.12154 | -0.17018 |
| C35 | 1.53167 | -0.05731 | -0.27522 |
| C36 | 1.50743 | -0.00705 | -0.30977 |
| C37 | 1.4392 | -0.02325 | -0.2307 |
| C38 | 1.39199 | -0.08812 | -0.13198 |
| C39 | 1.54909 | 0.05769 | -0.47905 |
| C40 | 1.50554 | -0.66548 | -0.25208 |
| C41 | 1.55567 | -0.6642 | -0.07121 |
| C42 | 1.54028 | 0.11538 | -0.44982 |
| O43 | 0.90466 | -0.43907 | 0.36658 |
| O44 | 1.02585 | -0.62003 | 0.24717 |
| O45 | 1.52435 | -0.44559 | 0.08586 |
| O46 | 1.35502 | -0.70728 | -0.37786 |
| O47 | 1.59774 | -0.04816 | -0.35043 |
| O48 | 1.32508 | -0.09564 | -0.07351 |
| C49 | 1.52194 | -0.3848 | -0.02081 |
| C50 | 1.35695 | -0.76396 | -0.22352 |
| O51 | 0.85523 | -0.34373 | 0.48196 |
| C52 | 0.8593 | -0.38432 | 0.72062 |
| C53 | 0.85786 | -0.44984 | 0.60276 |
| O54 | 1.09639 | -0.70992 | 0.12065 |
| C55 | 1.12307 | -0.65246 | 0.31639 |
| C56 | 1.09927 | -0.59861 | 0.24882 |
| O57 | 1.26909 | -0.85302 | -0.51256 |
| C58 | 1.29026 | -0.82902 | -0.22466 |
| O59 | 1.74051 | -0.00701 | -0.44936 |
| C60 | 1.71669 | 0.00485 | -0.17875 |
| C61 | 1.65297 | 0.01413 | -0.24049 |
| O62 | 1.17351 | -0.13242 | -0.18684 |
| C63 | 1.19774 | -0.16151 | 0.03901 |
| C64 | 1.26586 | -0.16141 | -0.0376 |
| O65 | 1.64522 | -0.31451 | -0.07153 |
| C66 | 1.58316 | -0.31898 | 0.0616 |
| C67 | 1.06099 | -0.77532 | 0.26397 |
| C68 | 1.10529 | -0.80248 | 0.4214 |
| O69 | 1.16126 | -0.8041 | 0.26222 |
| C70 | 1.21648 | -0.73625 | 0.2348 |
| C71 | 1.22291 | -0.92524 | -0.52492 |
| C72 | 1.25814 | -0.97178 | -0.51585 |
| O73 | 1.30653 | -0.96276 | -0.74411 |
| C74 | 1.3748 | -0.90771 | -0.72179 |
| C75 | 1.7083 | -0.2669 | 0.06699 |
| C76 | 1.7384 | -0.19323 | -0.04005 |
| O77 | 1.68863 | -0.16783 | -0.08722 |
| C78 | 1.65598 | -0.1629 | 0.17344 |
| C79 | 1.80235 | -0.01533 | -0.43416 |
| C80 | 1.86635 | 0.05095 | -0.39425 |
| O81 | 1.87411 | 0.10249 | -0.60272 |
| C82 | 1.83582 | 0.14081 | -0.55308 |
| C83 | 1.12023 | -0.1123 | -0.11871 |
| C84 | 1.05072 | -0.16719 | -0.02073 |
| O85 | 1.01208 | -0.22111 | -0.22079 |
| C86 | 1.04193 | -0.26709 | -0.26589 |
| C87 | 0.82235 | -0.30057 | 0.54627 |
| C88 | 0.74448 | -0.33701 | 0.49533 |
| O89 | 0.72666 | -0.38946 | 0.2778 |
| C90 | 0.70144 | -0.45818 | 0.38465 |
| H91 | 0.79325 | -0.69645 | 0.10571 |
| H92 | 0.7904 | -0.5575 | 0.25936 |
| H93 | 0.89769 | -0.65513 | 0.2508 |
| H94 | 1.03225 | -0.4024 | 0.3393 |
| H95 | 1.12876 | -0.48373 | 0.03731 |
| H96 | 1.15098 | -0.38736 | 0.57414 |
| H97 | 1.4461 | -0.23155 | 0.24844 |
| H98 | 1.30203 | -0.5387 | 0.23441 |
| H99 | 1.32081 | -0.23606 | -0.1063 |
| H100 | 1.40091 | -0.43533 | -0.22657 |
| H101 | 1.31696 | -0.61261 | -0.29188 |
| H102 | 1.56344 | -0.53585 | -0.02761 |
| H103 | 1.5056 | -0.16043 | -0.15155 |
| H104 | 1.41862 | 0.01476 | -0.26096 |
| H105 | 1.54919 | 0.03951 | -0.69929 |
| H106 | 1.48724 | -0.70227 | -0.43244 |
| H107 | 1.57609 | -0.62762 | 0.10662 |
| H108 | 1.52316 | 0.13519 | -0.63299 |
| H109 | 1.47688 | -0.3813 | 0.07623 |
| H110 | 1.51604 | -0.3878 | -0.25949 |
| H111 | 1.37336 | -0.74907 | 0.0047 |
| H112 | 1.39551 | -0.77718 | -0.32747 |
| H113 | 0.81481 | -0.39821 | 0.86983 |
| H114 | 0.90664 | -0.35308 | 0.84919 |
| H115 | 0.80515 | -0.48142 | 0.51979 |
| H116 | 0.86651 | -0.48059 | 0.77357 |
| H117 | 1.18047 | -0.62746 | 0.29912 |
| H118 | 1.11008 | -0.67001 | 0.54132 |
| H119 | 1.11972 | -0.57851 | 0.03071 |
| H120 | 1.12417 | -0.55808 | 0.41414 |
| H121 | 1.30042 | -0.86761 | -0.10572 |
| H122 | 1.24891 | -0.82173 | -0.10911 |
| H123 | 1.7564 | 0.05027 | -0.06502 |
| H124 | 1.70239 | -0.04057 | -0.03771 |
| H125 | 1.66616 | 0.05431 | -0.40924 |
| H126 | 1.63566 | 0.03166 | -0.04453 |
| H127 | 1.16011 | -0.21648 | 0.07583 |
| H128 | 1.2049 | -0.13341 | 0.24493 |
| H129 | 1.25837 | -0.19128 | -0.24031 |
| H130 | 1.27679 | -0.1888 | 0.14302 |
| H131 | 1.57184 | -0.27597 | -0.01171 |
| H132 | 1.58679 | -0.31803 | 0.30292 |
| H133 | 1.03068 | -0.81179 | 0.092 |
| H134 | 1.02422 | -0.77218 | 0.42331 |
| H135 | 1.0717 | -0.85633 | 0.48592 |
| H136 | 1.12492 | -0.77364 | 0.62635 |
| H137 | 1.22738 | -0.70682 | 0.44143 |
| H138 | 1.20425 | -0.70737 | 0.06466 |
| H139 | 1.26283 | -0.74116 | 0.16657 |
| H140 | 1.19558 | -0.93296 | -0.73427 |
| H141 | 1.18285 | -0.94033 | -0.35339 |
| H142 | 1.21867 | -1.02566 | -0.53676 |
| H143 | 1.2831 | -0.96852 | -0.30616 |
| H144 | 1.39028 | -0.88665 | -0.94248 |
| H145 | 1.40996 | -0.92797 | -0.64068 |
| H146 | 1.37799 | -0.8653 | -0.58062 |
| H147 | 1.7462 | -0.28728 | 0.02695 |
| H148 | 1.70113 | -0.26851 | 0.30528 |
| H149 | 1.76374 | -0.18899 | -0.25285 |
| H150 | 1.77978 | -0.1597 | 0.11152 |
| H151 | 1.63953 | -0.20988 | 0.30904 |
| H152 | 1.60977 | -0.15695 | 0.11013 |
| H153 | 1.68985 | -0.11864 | 0.30669 |
| H154 | 1.80604 | -0.03743 | -0.6457 |
| H155 | 1.79941 | -0.05289 | -0.26414 |
| H156 | 1.91157 | 0.0405 | -0.42333 |
| H157 | 1.86988 | 0.07079 | -0.17246 |
| H158 | 1.77921 | 0.10917 | -0.52292 |
| H159 | 1.84362 | 0.17176 | -0.75113 |
| H160 | 1.8567 | 0.17564 | -0.36406 |
| H161 | 1.11304 | -0.08902 | -0.32129 |
| H162 | 1.13827 | -0.07112 | 0.04807 |
| H163 | 1.0174 | -0.14175 | 0.0269 |
| H164 | 1.05759 | -0.18882 | 0.18608 |
| H165 | 1.09071 | -0.24159 | -0.39275 |
| H166 | 1.00398 | -0.31122 | -0.39118 |
| H167 | 1.05443 | -0.28534 | -0.05943 |
| H168 | 0.84726 | -0.25618 | 0.3973 |
| H169 | 0.83501 | -0.27959 | 0.76952 |
| H170 | 0.7253 | -0.30094 | 0.41217 |
| H171 | 0.71453 | -0.35811 | 0.69662 |
| H172 | 0.71662 | -0.48642 | 0.22338 |
| H173 | 0.64468 | -0.48114 | 0.40884 |
| H174 | 0.72379 | -0.46195 | 0.59581 |

**Table S2.** Fractional atomic coordinates for the eclipsed (AA) stacking unit cell of COF-O(2).

Crystal system: Hexagonal

Space group: P1

International tables number: 1

a= 21.4128 Å, b= 21.4128 Å, c= 3.5948 Å

α= β= 90^o^, γ= 120^o^

| Atom | x/a | y/b | z/c |
| --- | --- | --- | --- |
| N1 | 0.6768 | -0.71398 | 0.04228 |
| C2 | 0.70704 | -0.75337 | -0.0254 |
| N3 | 0.77102 | -0.72846 | -0.18418 |
| C4 | 0.80317 | -0.65987 | -0.28801 |
| N5 | 0.77849 | -0.61531 | -0.21582 |
| C6 | 0.71463 | -0.64511 | -0.05206 |
| C7 | 0.87735 | -0.62873 | -0.43254 |
| C8 | 0.9193 | -0.5591 | -0.53743 |
| C9 | 0.99884 | -0.52774 | -0.57011 |
| C10 | 1.02548 | -0.57378 | -0.643 |
| C11 | 1.09688 | -0.55245 | -0.57866 |
| C12 | 1.14278 | -0.48098 | -0.46454 |
| C13 | 1.1179 | -0.43221 | -0.43965 |
| C14 | 1.0454 | -0.45476 | -0.48141 |
| C15 | 1.21682 | -0.46036 | -0.33508 |
| C16 | 1.26355 | -0.39616 | -0.17993 |
| C17 | 1.33069 | -0.38347 | 0.00414 |
| N18 | 1.3377 | -0.44028 | 0.08344 |
| C19 | 1.39703 | -0.42604 | 0.26506 |
| N20 | 1.45021 | -0.3598 | 0.33948 |
| C21 | 1.43809 | -0.30689 | 0.25577 |
| N22 | 1.37851 | -0.31621 | 0.09094 |
| C23 | 1.41163 | -0.48576 | 0.33042 |
| C24 | 1.49785 | -0.23222 | 0.3132 |
| C25 | 1.4863 | -0.17592 | 0.29708 |
| C26 | 1.47157 | -0.47256 | 0.51804 |
| C27 | 1.54364 | -0.09866 | 0.29808 |
| C28 | 1.50691 | -0.51667 | 0.48228 |
| C29 | 1.46872 | -0.59193 | 0.43544 |
| C30 | 1.50931 | -0.62492 | 0.37575 |
| C31 | 1.5839 | -0.5851 | 0.33822 |
| C32 | 1.62181 | -0.5097 | 0.3846 |
| C33 | 1.58141 | -0.47755 | 0.4663 |
| C34 | 1.62078 | -0.62599 | 0.2378 |
| C35 | 1.68387 | -0.59866 | 0.05086 |
| C36 | 1.61293 | -0.07746 | 0.17332 |
| C37 | 1.66348 | -0.00508 | 0.12967 |
| C38 | 1.64194 | 0.04581 | 0.21176 |
| C39 | 1.57409 | 0.02412 | 0.36043 |
| C40 | 1.52398 | -0.04781 | 0.40186 |
| C41 | 1.6907 | 0.12381 | 0.1216 |
| C42 | 1.66481 | 0.16969 | 0.09001 |
| O43 | 1.01703 | -0.40966 | -0.42314 |
| O44 | 1.12244 | -0.60039 | -0.60907 |
| O45 | 1.73312 | 0.01892 | 0.00204 |
| O46 | 1.45566 | -0.07048 | 0.54121 |
| O47 | 1.39394 | -0.62982 | 0.44932 |
| O48 | 1.69638 | -0.46935 | 0.35147 |
| C49 | 1.06379 | -0.34013 | -0.26828 |
| C50 | 1.07346 | -0.67031 | -0.46423 |
| C51 | 1.41846 | -0.03808 | 0.37282 |
| C52 | 1.75183 | -0.03333 | -0.11545 |
| C53 | 1.35948 | -0.70039 | 0.29021 |
| C54 | 1.72145 | -0.39799 | 0.21555 |
| C55 | 1.02445 | -0.30151 | -0.15526 |
| C56 | 1.11147 | -0.70974 | -0.33724 |
| C57 | 1.28146 | -0.72883 | 0.1981 |
| C58 | 1.79765 | -0.35798 | 0.07467 |
| C59 | 1.33987 | -0.09425 | 0.3297 |
| C60 | 1.82853 | 0.00157 | -0.24951 |
| O61 | 1.07675 | -0.23291 | -0.01861 |
| O62 | 1.0662 | -0.77366 | -0.1328 |
| O63 | 1.25527 | -0.79904 | 0.05187 |
| O64 | 1.80445 | -0.29293 | -0.06595 |
| O65 | 1.84146 | -0.05357 | -0.3773 |
| O66 | 1.29788 | -0.06624 | 0.48081 |
| C67 | 1.01716 | -0.83226 | -0.35519 |
| C68 | 1.18006 | -0.83773 | -0.00678 |
| C69 | 1.87388 | -0.23664 | -0.16103 |
| C70 | 1.91437 | -0.02991 | -0.46234 |
| C71 | 1.22379 | -0.1183 | 0.47741 |
| C72 | 1.04942 | -0.1862 | 0.05215 |
| H73 | 0.89706 | -0.66641 | -0.40587 |
| H74 | 0.89996 | -0.52064 | -0.54078 |
| H75 | 0.98885 | -0.62958 | -0.7294 |
| H76 | 1.15516 | -0.37639 | -0.35786 |
| H77 | 1.229 | -0.50418 | -0.3429 |
| H78 | 1.25517 | -0.35034 | -0.1592 |
| H79 | 1.37755 | -0.53446 | 0.1725 |
| H80 | 1.55019 | -0.22773 | 0.35464 |
| H81 | 1.43194 | -0.18432 | 0.26304 |
| H82 | 1.5055 | -0.42042 | 0.65173 |
| H83 | 1.48238 | -0.6837 | 0.34187 |
| H84 | 1.60788 | -0.41891 | 0.50366 |
| H85 | 1.59052 | -0.68293 | 0.31459 |
| H86 | 1.71562 | -0.54336 | -0.04519 |
| H87 | 1.62605 | -0.11886 | 0.09051 |
| H88 | 1.55773 | 0.06308 | 0.44434 |
| H89 | 1.74655 | 0.13971 | 0.05874 |
| H90 | 1.60893 | 0.15394 | 0.14331 |
| H91 | 1.10584 | -0.30617 | -0.47032 |
| H92 | 1.09174 | -0.34538 | -0.02435 |
| H93 | 1.03255 | -0.70228 | -0.67463 |
| H94 | 1.04403 | -0.6654 | -0.22591 |
| H95 | 1.44488 | -0.01107 | 0.11224 |
| H96 | 1.42203 | 0.0046 | 0.55694 |
| H97 | 1.71522 | -0.06652 | -0.33854 |
| H98 | 1.74483 | -0.07057 | 0.11228 |
| H99 | 1.36295 | -0.73863 | 0.48224 |
| H100 | 1.38757 | -0.70037 | 0.03443 |
| H101 | 1.71738 | -0.36415 | 0.43354 |
| H102 | 1.68631 | -0.39967 | -0.01185 |
| H103 | 0.98311 | -0.33383 | 0.05454 |
| H104 | 0.99509 | -0.29698 | -0.39511 |
| H105 | 1.15487 | -0.67396 | -0.14576 |
| H106 | 1.13825 | -0.72018 | -0.57128 |
| H107 | 1.27645 | -0.69235 | -0.00129 |
| H108 | 1.2516 | -0.72954 | 0.44823 |
| H109 | 1.80609 | -0.38945 | -0.1423 |
| H110 | 1.83666 | -0.34848 | 0.29754 |
| H111 | 1.331 | -0.14277 | 0.48276 |
| H112 | 1.32395 | -0.11216 | 0.04183 |
| H113 | 1.86528 | 0.03295 | -0.02196 |
| H114 | 1.83666 | 0.04083 | -0.46973 |
| H115 | 0.97971 | -0.82054 | -0.51023 |
| H116 | 0.98534 | -0.87719 | -0.16426 |
| H117 | 1.04498 | -0.84942 | -0.5531 |
| H118 | 1.168 | -0.88801 | -0.14872 |
| H119 | 1.16176 | -0.8079 | -0.18242 |
| H120 | 1.14933 | -0.85173 | 0.25476 |
| H121 | 1.86546 | -0.19833 | -0.32682 |
| H122 | 1.90465 | -0.25523 | -0.32694 |
| H123 | 1.90552 | -0.20822 | 0.08651 |
| H124 | 1.93842 | 0.01743 | -0.64723 |
| H125 | 1.91506 | -0.07499 | -0.60465 |
| H126 | 1.94827 | -0.01639 | -0.2117 |
| H127 | 1.21189 | -0.16762 | 0.63149 |
| H128 | 1.20279 | -0.1342 | 0.19369 |
| H129 | 1.19524 | -0.09358 | 0.61075 |
| H130 | 1.00215 | -0.2109 | 0.23669 |
| H131 | 1.03457 | -0.16862 | -0.20401 |
| H132 | 1.09254 | -0.13813 | 0.18982 |

**Table S3.** Fractional atomic coordinates for the eclipsed (AA) stacking unit cell of COF-O(1).

Crystal system: Hexagonal

Space group: P1

International tables number: 1

a= 21.3888 Å, b= 21.3892 Å, c= 3.3500 Å

α= β= 90^o^, γ= 120^o^

| Atom | x/a | y/b | z/c |
| --- | --- | --- | --- |
| N1 | 0.67255 | -0.70927 | -0.54972 |
| C2 | 0.69784 | -0.75402 | -0.50854 |
| N3 | 0.76222 | -0.73523 | -0.35853 |
| C4 | 0.80324 | -0.66647 | -0.26693 |
| N5 | 0.78339 | -0.61737 | -0.30911 |
| C6 | 0.71891 | -0.64059 | -0.46625 |
| C7 | 0.87331 | -0.64476 | -0.07607 |
| C8 | 0.91463 | -0.57877 | 0.08733 |
| C9 | 0.98787 | -0.55353 | 0.2564 |
| C10 | 1.0133 | -0.60168 | 0.29533 |
| C11 | 1.08365 | -0.57813 | 0.407 |
| C12 | 1.12839 | -0.50407 | 0.48244 |
| C13 | 1.10152 | -0.45676 | 0.47068 |
| C14 | 1.03078 | -0.48051 | 0.36416 |
| C15 | 1.20681 | -0.47611 | 0.54641 |
| C16 | 1.25692 | -0.40525 | 0.53971 |
| C17 | 1.33395 | -0.38093 | 0.47653 |
| N18 | 1.3509 | -0.43104 | 0.38538 |
| C19 | 1.41764 | -0.40695 | 0.26152 |
| N20 | 1.46593 | -0.33773 | 0.2157 |
| C21 | 1.44359 | -0.292 | 0.30041 |
| N22 | 1.37703 | -0.31108 | 0.41303 |
| C23 | 1.43736 | -0.45982 | 0.11327 |
| C24 | 1.49276 | -0.21654 | 0.18444 |
| C25 | 1.46946 | -0.17152 | 0.06267 |
| C26 | 1.50074 | -0.43716 | -0.07802 |
| C27 | 1.51924 | -0.09662 | -0.07989 |
| C28 | 1.52889 | -0.48382 | -0.22907 |
| C29 | 1.48483 | -0.55902 | -0.28008 |
| C30 | 1.51826 | -0.597 | -0.39762 |
| C31 | 1.59206 | -0.56231 | -0.467 |
| C32 | 1.63541 | -0.48669 | -0.43544 |
| C33 | 1.60191 | -0.4488 | -0.32041 |
| C34 | 1.62369 | -0.60878 | -0.5578 |
| C35 | 1.6918 | -0.58995 | -0.45407 |
| C36 | 1.59244 | -0.07295 | -0.12463 |
| C37 | 1.64075 | -0.0026 | -0.22885 |
| C38 | 1.61282 | 0.04313 | -0.31065 |
| C39 | 1.53941 | 0.01908 | -0.27895 |
| C40 | 1.49138 | -0.0508 | -0.16257 |
| C41 | 1.66446 | 0.11999 | -0.40539 |
| C42 | 1.64534 | 0.16856 | -0.5207 |
| O43 | 1.00184 | -0.4351 | 0.34284 |
| O44 | 1.11158 | -0.62438 | 0.42121 |
| O45 | 1.71469 | 0.02408 | -0.25771 |
| O46 | 1.41772 | -0.07665 | -0.13122 |
| O47 | 1.41081 | -0.59251 | -0.22364 |
| O48 | 1.70845 | -0.4523 | -0.52621 |
| C49 | 1.0364 | -0.36784 | 0.54829 |
| C50 | 1.0641 | -0.69576 | 0.5586 |
| C51 | 1.3963 | -0.02663 | -0.00532 |
| C52 | 1.74612 | -0.0119 | -0.05401 |
| C53 | 1.37369 | -0.66615 | -0.10407 |
| C54 | 1.757 | -0.38809 | -0.3174 |
| H55 | 0.88751 | -0.68679 | -0.0887 |
| H56 | 0.89753 | -0.53845 | 0.08676 |
| H57 | 0.97902 | -0.6588 | 0.22054 |
| H58 | 1.13736 | -0.39924 | 0.52929 |
| H59 | 1.22248 | -0.51751 | 0.54407 |
| H60 | 1.24545 | -0.36132 | 0.52676 |
| H61 | 1.39742 | -0.51545 | 0.17837 |
| H62 | 1.54864 | -0.20355 | 0.18563 |
| H63 | 1.4122 | -0.18802 | 0.06002 |
| H64 | 1.53787 | -0.3796 | -0.12349 |
| H65 | 1.48636 | -0.65585 | -0.42987 |
| H66 | 1.63362 | -0.3898 | -0.29577 |
| H67 | 1.58588 | -0.66226 | -0.67444 |
| H68 | 1.72967 | -0.53899 | -0.31494 |
| H69 | 1.61314 | -0.10984 | -0.07567 |
| H70 | 1.51879 | 0.05581 | -0.33873 |
| H71 | 1.72062 | 0.13662 | -0.33827 |
| H72 | 1.59083 | 0.1565 | -0.58808 |
| H73 | 1.0677 | -0.32339 | 0.3392 |
| H74 | 1.07297 | -0.36646 | 0.7818 |
| H75 | 0.99489 | -0.35921 | 0.68939 |
| H76 | 1.09531 | -0.71384 | 0.73654 |
| H77 | 1.03947 | -0.73305 | 0.30723 |
| H78 | 1.02061 | -0.69822 | 0.74432 |
| H79 | 1.3465 | -0.05567 | 0.1713 |
| H80 | 1.38533 | -0.00179 | -0.26197 |
| H81 | 1.43816 | 0.01659 | 0.17809 |
| H82 | 1.75805 | -0.044 | -0.26397 |
| H83 | 1.71062 | -0.04798 | 0.18017 |
| H84 | 1.79685 | 0.02875 | 0.08609 |
| H85 | 1.40841 | -0.67916 | 0.07742 |
| H86 | 1.35513 | -0.70218 | -0.3635 |
| H87 | 1.32615 | -0.67631 | 0.07225 |
| H88 | 1.78423 | -0.3428 | -0.52696 |
| H89 | 1.79807 | -0.39651 | -0.16848 |
| H90 | 1.72983 | -0.3733 | -0.08991 |

**Table S4.** Fractional atomic coordinates for the eclipsed (AA) stacking unit cell of COF-O(0).

Crystal system: Hexagonal

Space group: P1

International tables number: 1

a= 21.5743 Å, b= 21.5783 Å, c= 3.3999 Å

α= β= 90^o^, γ= 120^o^

| Atom | x/a | y/b | z/c |
| --- | --- | --- | --- |
| N1 | 0.64602 | -0.70729 | 0.47698 |
| C2 | 0.67205 | -0.7512 | 0.48755 |
| N3 | 0.74138 | -0.72961 | 0.47468 |
| C4 | 0.78532 | -0.65968 | 0.48364 |
| N5 | 0.76371 | -0.61195 | 0.47311 |
| C6 | 0.69378 | -0.63796 | 0.48593 |
| C7 | 0.86139 | -0.63441 | 0.39341 |
| C8 | 0.90715 | -0.56659 | 0.2767 |
| C9 | 0.98148 | -0.54125 | 0.13636 |
| C10 | 1.00804 | -0.5885 | 0.09216 |
| C11 | 1.07683 | -0.56346 | -0.04452 |
| C12 | 1.1201 | -0.49106 | -0.14293 |
| C13 | 1.09355 | -0.4438 | -0.09863 |
| C14 | 1.02475 | -0.46885 | 0.03792 |
| C15 | 1.1944 | -0.46572 | -0.28339 |
| C16 | 1.24017 | -0.39788 | -0.39955 |
| C17 | 1.31633 | -0.3725 | -0.48787 |
| N18 | 1.33795 | -0.42022 | -0.4772 |
| C19 | 1.40789 | -0.39418 | -0.48694 |
| N20 | 1.45561 | -0.32484 | -0.47494 |
| C21 | 1.42958 | -0.28094 | -0.48575 |
| N22 | 1.36023 | -0.30257 | -0.47588 |
| C23 | 1.43321 | -0.44498 | -0.39767 |
| C24 | 1.48031 | -0.20487 | -0.3957 |
| C25 | 1.45817 | -0.15919 | -0.27885 |
| C26 | 1.50096 | -0.4229 | -0.27947 |
| C27 | 1.50708 | -0.08493 | -0.13783 |
| C28 | 1.52623 | -0.47185 | -0.1382 |
| C29 | 1.47892 | -0.54565 | -0.09354 |
| C30 | 1.50393 | -0.58937 | 0.0438 |
| C31 | 1.57633 | -0.56025 | 0.14248 |
| C32 | 1.62362 | -0.48644 | 0.09815 |
| C33 | 1.59862 | -0.44272 | -0.03929 |
| C34 | 1.60167 | -0.60923 | 0.28285 |
| C35 | 1.66957 | -0.58718 | 0.39791 |
| C36 | 1.58091 | -0.05836 | -0.09346 |
| C37 | 1.62463 | 0.01041 | 0.04337 |
| C38 | 1.59545 | 0.05363 | 0.14197 |
| C39 | 1.52162 | 0.02705 | 0.09785 |
| C40 | 1.47791 | -0.0417 | -0.0392 |
| C41 | 1.64443 | 0.12797 | 0.28166 |
| C42 | 1.62238 | 0.17363 | 0.40028 |
| H43 | 0.87471 | -0.67667 | 0.40539 |
| H44 | 0.89072 | -0.52632 | 0.26289 |
| H45 | 0.97583 | -0.64551 | 0.16364 |
| H46 | 1.09672 | -0.60114 | -0.07473 |
| H47 | 1.12576 | -0.38679 | -0.17005 |
| H48 | 1.00486 | -0.43117 | 0.06806 |
| H49 | 1.21085 | -0.50598 | -0.26962 |
| H50 | 1.22685 | -0.35561 | -0.4111 |
| H51 | 1.39097 | -0.50056 | -0.41053 |
| H52 | 1.53591 | -0.19153 | -0.40713 |
| H53 | 1.40144 | -0.17566 | -0.26535 |
| H54 | 1.54118 | -0.36619 | -0.26459 |
| H55 | 1.42191 | -0.57043 | -0.16526 |
| H56 | 1.46623 | -0.64693 | 0.07405 |
| H57 | 1.68063 | -0.46163 | 0.1699 |
| H58 | 1.63633 | -0.38517 | -0.06973 |
| H59 | 1.56136 | -0.66593 | 0.26969 |
| H60 | 1.71189 | -0.53161 | 0.40861 |
| H61 | 1.60574 | -0.09053 | -0.16517 |
| H62 | 1.68221 | 0.03033 | 0.07339 |
| H63 | 1.49682 | 0.05928 | 0.16925 |
| H64 | 1.42033 | -0.06159 | -0.06953 |
| H65 | 1.70117 | 0.14456 | 0.26517 |
| H66 | 1.56678 | 0.16019 | 0.41485 |

**Table S5.** Comparison of representative COF/MOF-based acidic OER electrocatalysts.

| Catalyst | Electrolyte | Potential at the 10 mA cm^-2^ (vs. RHE) | Tafel  slope (mV dec^-1^) | Ref. |
| --- | --- | --- | --- | --- |
| RuO_2_@COF-O(3) | **0.5 M H_2_SO_4_** | **218 mV** | **64.3** | **This work** |
| COF-205-Ru | 0.5 M H_2_SO_4_ | 212 mV | 70 | [6] |
| CoCl_2_@Th-BPYDC | 0.1 M HClO_4_ | 388 mV | 94 | [7] |
| Ir-UiO-66-10CNT | 0.1 M HClO_4_ | 430 mV | 139.7 | [8] |
| Ru-UiO-67-bpydc | 0.5 M H_2_SO_4_ | 200 mV | 78.3 | [9] |

**Table S6.** Summary of impedance fitting data for RuO_2_@COF-O(3), RuO_2_@COF-O(2), RuO_2_@COF-O(1), RuO_2_@COF-O(0), and RuO_2_.

| Samples | Rs (Ω) | Rp (Ω) | CPE_p_ (mMho*s^N (N)) |
| --- | --- | --- | --- |
| RuO_2_@COF-O(3) | 9.8 | 39.6 | 6.72 (0.737) |
| RuO_2_@COF-O(2) | 10.1 | 66.9 | 2.91 (0.726) |
| RuO_2_@COF-O(1) | 10.5 | 80.6 | 2.14 (0.777) |
| RuO_2_@COF-O(0) | 13.6 | 149.0 | 1.45 (0.727) |
| RuO_2_ | 13.5 | 266 | 0.784 (0.882) |

**Table S7.** ICP-MS results of leached Ru species for RuO_2_@COF-O(3) and RuO_2_ after OER stability test.

| Sample | RuO_2_ amount (mg) | Concentrations of Ru ion (μg mL^-1^) | Loss of mass Ru | |
| --- | --- | --- | --- | --- |
| RuO_2_@COF-O(3) | 0.28 | 0.029 | | 0.52 wt.% |
| RuO_2_ | 0.28 | 0.088 | | 1.57 wt.% |

**Table S8.** Peak assignments of Raman simulation for COF-O(3).

| Peak position (cm^-1^) | Definition |
| --- | --- |
| 1692 | Stretching vibration of C=C |
| 1656 | Stretching vibration of C=C (phenyl) |
| 1584 | Stretching vibration of C-N |
| 1302 | Stretching vibration of C-O |
| 1000 | Bending vibration of C-H |

**Table S9.** The deconvolution of broad feature of O-H stretching modes for RuO_2_@COF-O(3) at different applied potentials.

| Applied potential  (V vs. RHE) | Peak center | Area Intg | FWHM | Area IntgP (% ) |
| --- | --- | --- | --- | --- |
| 1.2 | 3500.01 | 0.097 | 209.74 | 35.22 |
|  | 3303.21 | 0.179 | 369.71 | 64.77 |
| 1.3 | 3512.99 | 0.347 | 220.00 | 35.51 |
|  | 3322.76 | 0.631 | 364.44 | 64.48 |
| 1.4 | 3511.09 | 0.622 | 206.14 | 35.90 |
|  | 3314.38 | 1.110 | 352.88 | 64.11 |
| 1.5 | 3515.93 | 0.877 | 213.18 | 34.78 |
|  | 3317.31 | 1.644 | 363.58 | 65.22 |
| 1.6 | 3510.95 | 1.213 | 196.93 | 36.08 |
|  | 3313.89 | 2.150 | 338.10 | 63.92 |
| 1.7 | 3511.12 | 1.437 | 201.20 | 35.38 |
|  | 3312.01 | 2.626 | 348.22 | 64.62 |
| 1.8 | 3510.35 | 1.802 | 198.44 | 35.57 |
|  | 3309.65 | 3.264 | 343.32 | 64.43 |

FWHM: full width at half maximum

Area Intg: peak area by integrating data

Area IntgP: a percent of integrated peak area

**Table S10.** The deconvolution of broad feature of O-H stretching modes for RuO_2_ at different applied potentials.

| Applied potential  (V vs. RHE) | Peak center | Area Intg | FWHM | Area IntgP (% ) |
| --- | --- | --- | --- | --- |
| 1.2 | 3467.72 | 0.122 | 238.41 | 58.72 |
|  | 3264.51 | 0.086 | 247.62 | 41.28 |
| 1.3 | 3468.57 | 0.265 | 240.79 | 56.73 |
|  | 3263.13 | 0.202 | 263.53 | 43.27 |
| 1.4 | 3467.17 | 0.414 | 239.33 | 57.39 |
|  | 3262.21 | 0.307 | 256.43 | 42.61 |
| 1.5 | 3463.51 | 0.623 | 244.59 | 60.11 |
|  | 3258.46 | 0.413 | 248.96 | 39.89 |
| 1.6 | 3454.71 | 0.842 | 244.05 | 60.50 |
|  | 3249.21 | 0.550 | 223.00 | 39.51 |
| 1.7 | 3447.90 | 1.008 | 259.74 | 63.54 |
|  | 3235.89 | 0.579 | 223.85 | 36.46 |
| 1.8 | 3432.98 | 1.152 | 263.52 | 65.48 |
|  | 3228.17 | 0.607 | 215.15 | 34.51 |

FWHM: full width at half maximum

Area Intg: peak area by integrating data

Area IntgP: a percent of integrated peak area

**Supplementary Note 1.** Calculation of the energy consumption to produce 1 kg H_2_ at a current density of 1.0 A cm^-2^.

The energy consumption (kWh/kg_H2_) is calculated by the following equation:

Energy consumption =
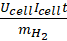


where 𝐼_cell_ is the current (A), t is the operation time (h), m_H2_ is the mass of hydrogen produced in a t duration, which can be calculated by the Faraday's laws of electrolysis:


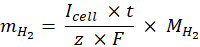


z is the number of electrons transferred to produce one hydrogen molecule, M_H2_ relative molecular mass (2 g mol^-1^).

**Table S11. Comparisons of the PEMWE activity and stability with those previously reported.**

| Catalyst layer | Electrolyzer | Cell voltage /V | Operation time /h | Loading amount of anodic noble metal cost | Anode noble metal cost /$ cm^-2^ | Ref. |
| --- | --- | --- | --- | --- | --- | --- |
| **RuO_2_@COF-O(3)** | **distilled water** | **1.54@1 A cm^−2^** | **>180@0.2 A cm^-2^** | **0.76 mg_Ru_ cm^-2^** | **0.0109** | **This work** |
| Nb_0.1_Ru_0.9_O_2_ | DI water | 1.69@1 A cm^−2^ | 100@0.3 A cm^-2^ | 1.6 mg_Ru_ cm^-2^ | 0.022 | [10] |
| RuO_2_/SnO_2_ 1.5X | pure water | 1.61@1 A cm^−2^ | >25@0.5 A cm^-2^ | 0.3 mg_Ru_ cm^-2^ | 0.01143 | [11] |
| Ni-RuO_2_ | 0.1 M HClO_4_ | 1.65@1 A cm^−2^ | 1000@0.2 A cm^-2^ | ~2.3  mg_Ru_ cm^-2^ | 0.032 | [12] |
| RuO_2_-NS/CF | deionized water | 1.57@1 A cm^−2^ | 10@1 A cm^-2^ | 0.9 mg_Ru_ cm^-2^ | 0.0127 | [13] |
| IrO_2_-RuO_2_-TaO_x_ | pH (<2) | ~1.651@1A cm^−2^ | NA | 1 mg_Ir + Ru_ cm^−2^ | 0.0874 | [14] |
| MEA Ir-Ru 1:3 | NA | ~1.68@1 A cm^−2^ | >90@1 A cm^-2^ | 0.07 mg_Ir + Ru_ cm^−2^ | 0.00355 | [15] |
| Ru_0.5_Ir_0.5_O_2_ | 0.5 M H_2_SO_4_ | 1.605@1 A cm^−2^ | 255@200 mA cm^-2^ | 0.28 mg_Ru_ cm^-2^ | 0.0489 | [16] |
| (Ru-W)O_x_ | H_2_O | 1.62@1 A cm^−2^ | 300@0.5 A cm^−2^ | 0.08 mg_Ru_ cm^-2^ | 0.0008 | [17] |
| SrRuIr | H_2_O | 1.5@1 A cm^−2^ | 150@1 A cm^−2^ | 1.034 mg_Ir + Ru_ cm^−2^ | 0.032 | [18] |
| RuO_x_ | distilled water | 1.73@1 A cm^−2^ | 50@1 A cm^-2^ | 2 mg_Ru_ cm^-2^ | 0.055 | [19] |
| RuCoO_x_ | 0.5 M H_2_SO_4_ | 1.6@0.4 A cm^−2^ | 10@100 mA cm^-2^ | 0.085 mg_Ru_ cm^-2^ | 0.0012 | [20] |

The price of the noble metal (Ir and Ru) is obtained from the Johnson Matthey Price Charts.

**References**

[1] Y. Okada, M. Sugai, K. Chiba, *J. Org. Chem.* **2016**, *81*, 10922.

[2] a) Lu, T., Chen, Q., *J. Comput. Chem.* **2022**, *43*, 539; b) Lu, T. & Chen, F., *J. Comput. Chem.* **2012**, *33*, 580.

[3] S. Grimme, J. Antony, S. Ehrlich, H. Krieg, *J. Chem. Phys.* **2020***, 152*, 194103.

[4] Grimme, S., Bannwarth, C., Shushkov, P. *J. Chem. Theory. Comput.* **2017**, *13*, 1989.

[5] W. Humphrey, A. D., K. Schulten. *J. Mol. Graph.* **1996***, 14*, 33.

[6] H. Jia, N.Yao, Y. Jin, L. Wu, J. Zhu, W. Luo, *Nat. Commun.* **2024**, *15*, 5419.

[7] Z. Gao, Y. Lai, L. Gong, L. Zhang, S. Xi, J. Sun, L. Zhang, F. Luo, *ACS Catal.* **2022**, *12*, 9101-9113.

[8] T. Chang, C. Chuang, Y. Chen, Y. Wang, Y. Gu, and C. Kung, *ChemCatChem* **2022**, *14*, e202200199.

[9] N. Yao, H. Jia, J. Zhu, Z. Shi, H. Cong, J. Ge, W. Luo, *Chem* **2023**, *9*, 1882-1896.

[10] H. Liu, Z. Zhang, J. Fang, M. Li, M. G. Sendeku, X. Wang, H. Wu, Y. Li, J. Ge, Z. Zhuang, D. Zhou, Y. Kuang, X. Sun, *Joule* **2023**, *7*, 558.

[11] B. Huang, Y. Cui, X. Liu, C. Zheng, H. Wang, L. Guan, *Small* **2023**, *19*, e2301516.

[12] Z. Y. Wu, F. Y. Chen, B. Li, S. W. Yu, Y. Z. Finfrock, D. M. Meira, Q. Q. Yan, P. Zhu, M. X. Chen, T. W. Song, Z. Yin, H. W. Liang, S. Zhang, G. Wang, H. Wang, *Nat. Mater.* **2023**, *22*, 100.

[13] H. Huang, H. Kim, A. Lee, S. Kim, W.-G. Lim, C.-Y. Park, S. Kim, S.-K. Kim, J. Lee, *Nano Energy* **2021**, *88*, 106276.

[14] Z. Fan, H. Yu, G. Jiang, D. Yao, S. Sun, J. Chi, B. Qin, Z. Shao, *Int. J. Hydrogen Energ.*  **2022**, *47*, 18963.

[15] T. Hrbek, P. Kúš, T. Košutová, K. Veltruská, T. N. Dinhová, M. Dopita, V. Matolín, I. Matolínová, *Int. J. Hydrogen Energ.* **2022**, *47*, 21033.

[16] W. Zhu, X. Song, F. Liao, H. Huang, Q. Shao, K. Feng, Y. Zhou, M. Ma, J. Wu, H. Yang, H. Yang, M. Wang, J. Shi, J. Zhong, T. Cheng, M. Shao, Y. Liu, Z. Kang, *Nat. Commun.* **2023**, *14*, 5365.

[17] L. Deng, S. F. Hung, Z. Y. Lin, Y. Zhang, C. Zhang, Y. Hao, S. Liu, C. H. Kuo, H. Y. Chen, J. Peng, J. Wang, S. Peng, *Adv Mater* **2023**, *35*, e2305939.

[18] Y. Wen, P. Chen, L. Wang, S. Li, Z. Wang, J. Abed, X. Mao, Y. Min, C. T. Dinh, P. Luna, R. Huang, L. Zhang, L. Wang, L. Wang, R. J. Nielsen, H. Li, T. Zhuang, C. Ke, O. Voznyy, Y. Hu, Y. Li, W. A. Goddard, III, B. Zhang, H. Peng, E. H. Sargent, *J. Am. Chem. Soc.* **2021**, *143*, 6482.

[19] Z. Shi, J. Li, Y. Wang, S. Liu, J. Zhu, J. Yang, X. Wang, J. Ni, Z. Jiang, L. Zhang, Y. Wang, C. Liu, W. Xing, J. Ge, *Nat. Commun.* **2023**, *14*, 843.

[20] W. Zhu, F. Yao, K. Cheng, M. Zhao, C. J. Yang, C. L. Dong, Q. Hong, Q. Jiang, Z. Wang, H. Liang, *J. Am. Chem. Soc.* **2023**, *145*, 17995.
